# Fisheries management tools to support coastal and marine spatial planning: A case study from the Northern Gulf of California, Mexico

**DOI:** 10.1016/j.mex.2020.101108

**Published:** 2020-10-16

**Authors:** Hem Nalini Morzaria-Luna, Peggy Turk-Boyer, José Manuel Dorantes Hernández, Elia Polanco-Mizquez, Caroline Downton-Hoffmann, Gabriela Cruz-Piñón, Tonatiuh Carrillo-Lammens, Rene Loaiza-Villanueva, Paloma Valdivia-Jiménez, Angeles Sánchez-Cruz, Valeria Peña-Mendoza, Ariadna Montserrat López-Ortiz, Volker Koch, Leonardo Vázquez-Vera, José Alfredo Arreola-Lizárraga, Imelda G. Amador-Castro, Alvin N. Suárez Castillo, Adrian Munguia-Vega

**Affiliations:** a CEDO Intercultural, Apartado Postal #53. Puerto Peñasco, Sonora, México C.P. 83550. / P.O. Box 44208, Tucson, AZ 85733, United States; b Visiting Scientist. Northwest Fisheries Science Center, NOAA. 2725 Montlake Blvd. Seattle WA 98112, United States; c Director Emeritus. CEDO Intercultural, P.O. Box 44208, Tucson, AZ 85733, United States; d Departamento de Ciencias Marinas y Costeras. Universidad Autónoma de Baja California Sur. Carretera al Sur Km 5.5, Apartado Postal 19-B, C.P., La Paz, Baja California Sur 23080, Mexico; e Deutsche Gesellschaft für Internationale Zusammenarbeit (GIZ) GmbH, P.O. Box 5180, Eschborn 65726, Germany; f Departamento de Ecología Marina, Centro de Investigación Científica y Educación Superior de Ensenada, Ensenada, Baja California, Mexico; g The Nature Conservancy, La Paz, Baja California Sur, 23000, Mexico; h Centro de Investigaciones Biológicas del Noroeste, S.C. Campus Guaymas, Km 2.3 carr. a Las Tinajas predio El Tular s/n, Guaymas, Sonora CP. 85454, Mexico; i Comunidad y Biodiversidad, A.C. Isla del Peruano 215, Lomas de Miramar, Guaymas,Sonora, CP. 85448, Mexico; j Desert Laboratory on Tumamoc Hill, University of Arizona, Tucson, AZ 85721, United States

**Keywords:** Atlantis ecosystem model, Vulnerability analysis, Froese sustainability indicators, Spatial prioritization, Coastal marine spatial planning

## Abstract

A management approach was developed that combined spatial and non-spatial tools to inform a Coastal and Marine Spatial Planning Process (CMSP) in the Puerto Peñasco-Puerto Lobos Coastal Corridor, Northern Gulf of California, Sonora, Mexico. Four fisheries management tools were applied with an emphasis on ecosystem level management for eleven small-scale fisheries. Two spatial management tools, using a spatial prioritization approach, were combined with a permit regularization process, a non-spatial quota prioritization, and a tradeoff analysis in a novel way:

• Locally Managed Marine Areas were developed, these are spatial areas where individual community fishermen are assigned the rights to harvest and manage specific fisheries within defined geographic areas.

• Fishery refuges that incorporate information on fisheries, ecological importance, and connectivity.

• A non-spatial quota prioritization process using a framework for the integrated assessment of stocks, encompassing a vulnerability analysis, a sustainability analysis, and a management framework analysis.

• A trade-off analysis of the combination of these different management tools, using an Atlantis ecosystem model for the northern Gulf of California, that tested the ecosystem effects of alternative scenarios to assess benefits in support of ecosystem-based management.

## Graphical abstract

**Figure:**
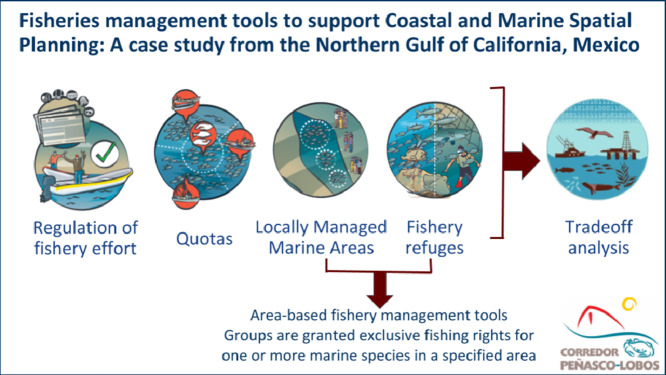

Specifications table

**Table:** 

Subject Area	Environmental Science
More specific subject area	Ecosystem-based management
Method name	*Coastal and Marine Spatial Planning, using spatial prioritization, ecosystem modeling*
Name and reference of original method	Morzaria-Luna, H., P. Turk-Boyer, E. Polanco-Mizquez, C. Downton-Hoffmann, G. Cruz-Piñón, T. Carrillo-Lammens, R. Loaiza-Villanueva, P. Valdivia-Jiménez, Á. Sánchez-Cruz, V. Peña-Mendoza, A. Montserrat López-Ortiz, V. Koch, L. Vázquez-Vera, J. Alfredo Arreola-Lizárraga, I. G. Amador-Castro, A.N. Suárez Castillo, A. Munguia-Vega. Coastal and Marine Spatial Planning in the Northern Gulf of California, Mexico: Consolidating Stewardship, Property Rights, and Enforcement for Ecosystem-Based Fisheries Management. Ocean and Coastal Management. Oceans and Coastal Management
Resource availability	Software: http://fishe.edf.org/get-started https://research.csiro.au/atlantis/

## Background

The increase in overexploitation of ocean areas due to resource extraction, habitat degradation and pollution, highlights the need for management approaches that address diverse impacts on marine resources [14]. Australia was one of the first countries to implement spatial planning strategies to resolve conflicts over use of marine resources; in 1975 the Great Barrier Reef Marine Park Act was promulgated to address concerns about oil drilling and limestone extraction. Using a wide variety of zoning tools, this Act led to the design,establishment and management of a 344,400 km^2^ extension of Australia's east coast marine ecosystem, as a multiple-use marine park [13,32]. Along the southern California coast, concerns about the decline of target species and oil exploration led to the 1980 establishment of the Channel Island National Marine Sanctuary. Through the planning process for this Sanctuary eleven marine reserves and two conservation areas where some fishing is permitted were created, offering protection for 21% of Sanctuary waters. These and other case studies have contributed to development of tools and approaches for ecosystem management; their successes can be attributed to organization and management, feedback from governance, public and scientific contributions to the process; and the planned use of recreational activities and sustainable economic uses [28,51].

In July 2010, the U.S. government, through Order 13,547, began using Coastal and Marine Spatial Planning (CMSP) as a policy tool [26]. The CMSP approach brings together all stakeholders that use a defined area to plan for the sustainable use of marine and coastal resources, while balancing diverse economic interests [35]. CMSP employs participatory decision-making approaches that use scientific and geospatial information to organize human activities in a sustainable and efficient way to address conflicts in different marine and coastal areas [11]. It involves identifying the most suitable areas for different activities, including sport fishing, ecological tourism, commercial services, among others; it seeks to reduce environmental problems by facilitating compatible uses and preserving critical ecosystem health, function, and services [17,51]. It has been challenging, however, to make the transition from planning to implementation, which is paramount for achieving sustainable ecosystem and governance outcomes [16].

A CMSP approach was developed and applied in the Northern Gulf of California, Sonora, Mexico that can be used as an ecosystem-level tool for solving some of the biggest challenges faced by coastal communities. This CMSP process relies on spatial prioritization of use; employing computational tools and analyses to allocate the spatial and temporal distribution of human activities in marine areas, in order to achieve ecological, economic and social objectives; it is supported by public processes with citizen participation, and based on a framework of managing current and future ecosystem use for future generations with the integration of ecological, economic and social sectors [15,^22^,^30^,34]. Spatial prioritization is an essential tool to resolve intersectoral and transboundary conflicts in maritime space; it can improve decision-making, strengthen property rights and stewardship (which are largely space-based), reduce conflicts between resource users, improve compliance with regulations, and enhance the government's commitment to enforcement; it offers an ecosystem-based approach for management [18,^19^,27].

From 2015 to 2019, four fisheries management tools were combined to advance CMSP in an eco-region known as the Penasco-Lobos Coastal Corridor. Two spatial management tools were used: (1) Locally Managed Marine Areas and (2) Fishery refuges; one non-spatial tool, (3) Catch quotas and (4) Regularization of fishing effort through permits with explicit spatial definition for the region. Finally, these tools were integrated through a Trade-off analysis to examine the ecosystem effects of scenarios representing different combinations of spatial and non-spatial tools. To develop the spatial management tools, spatial prioritization was used approach, that was developed for high-resolution, large-scale conservation; it has been applied in marine, riparian and terrestrial environments to identify refuge networks, zones for the expansion of affected areas; to evaluate marine protected areas; identify areas of lower ecological value; balance alternative uses; as well as to prioritize communities and species [10,33]. Right-based areas were explored where groups are granted exclusive fishing rights for one or more marine species in a specified area (similar to Territorial Use Rights for Fisheries- reserves or TURFs) to clarify property rights and control problems of open access [9]; called these ‘Locally Managed Marine Areas’. Then, an analysis was performed to determine prioritization of quota implementation for key fishery species. This prioritization was carried out using the framework for integrated assessment of stock and habitat (Fisheries toolbox - FISHE), developed by the Environmental Defense Fund, which combines a vulnerability analysis, Froese indicators of sustainability and the analysis of attributes based on management. These analyses were conducted within the framework of the actual fishing effort, including regular (permitted) and irregular (traditional) fishing, and separately a proposal was developed for clarifying property rights through permit regularization within the spatial boundaries of the corridor ecosystem.

Rights-based management areas have been implemented extensively in Japan and Chile coastal fisheries [56]. There are several successful examples of spatial management in Mexico as well. Fishing cooperatives in the Vizcaíno Peninsula, Baja California Sur, have exclusive access and use rights for abalone, lobster, conch, and other species, in defined areas of the Pacific coast under exclusive use concessions [37]. In Sonora, the Seri or Comcaác have territorial rights in their coastal territory where they manage the pen shell fishery within the Canal del Infiernillo [7]. While in Punta Allen, Quintana Roo, within the Sian Ka'an Biosphere Reserve, one fishing cooperative informally established spatial management areas that were later formalized through a concession [38]. Fishing refuges are codified in existing Mexican legislation (NOM-049-SAG / PESC-2014 [36]), as areas where fishing activities are prohibited or restricted and aim to contribute to the development of fishery resources and protect their habitat [31]. Fishing refuges in Mexico were created in 2007; there are only three examples at the national level so far. A network of 11 refuges covering 1409 ha in the Gulf of California, was established in the San Cosme-Punta Coyote fishing corridor in the municipality of La Paz. These refuges were established in areas that were considered over-exploited and their implementation involved the close participation of fishermen from neighboring communities and constant feedback between federal, state and local environmental authorities, guided by a non-government organization [21,55].

**Fig. 1:**
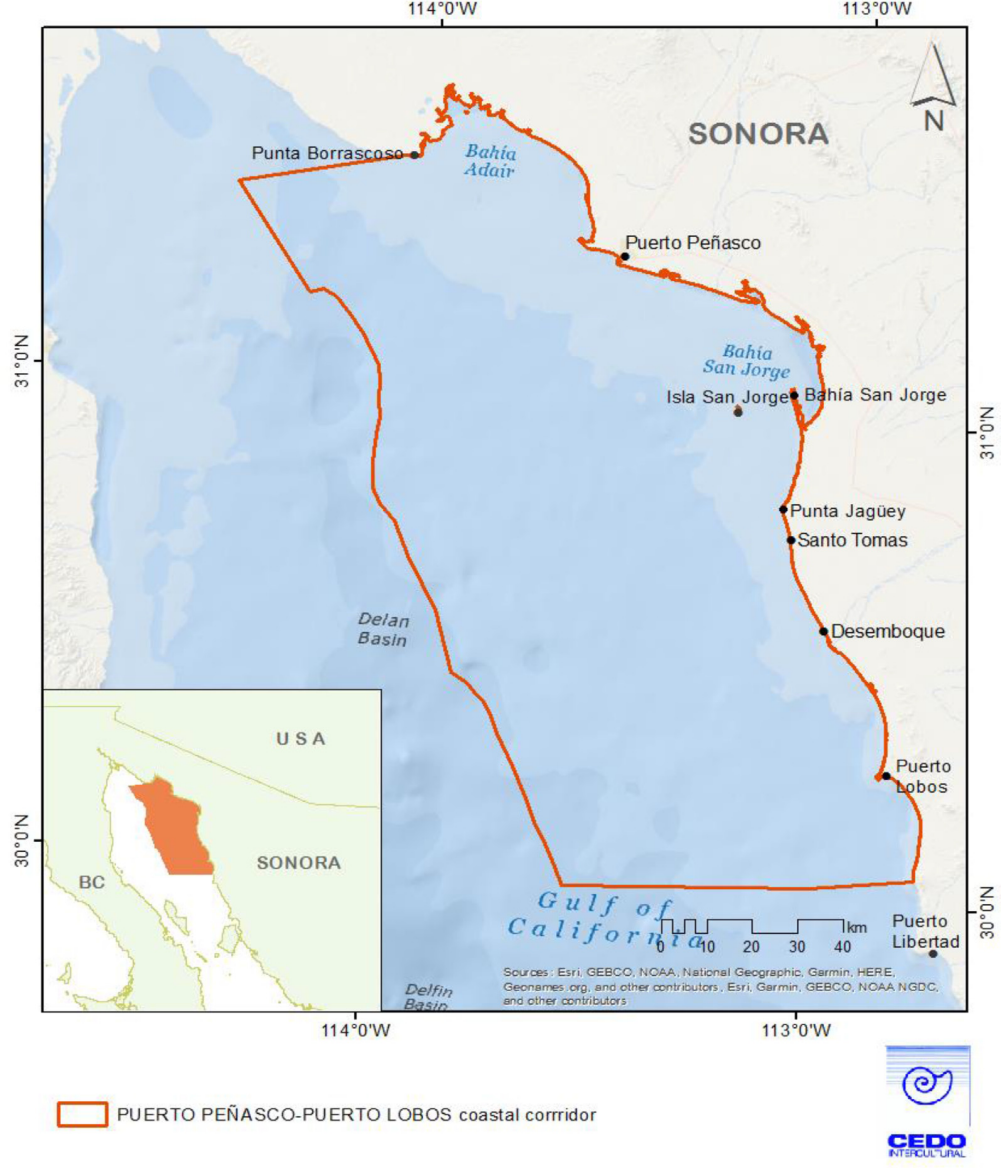
Puerto Peñasco-Puerto Lobos Coastal Corridor, Northern Gulf of California, Sonora, Mexico. The Corridor area incorporates the primary fishing areas of six communities, existing management areas, and marine and coastal habitats.

A CMSP approach was applied to a Coastal Corridor in the Northern Gulf of California, Sonora, with emphasis on management of eleven small-scale fisheries (Table 1). The Puerto Peñasco-Puerto Lobos Coastal Corridor is a 200-km stretch of coastline in the northeastern Gulf of California, Mexico (Fig. 1). There are six coastal communities within the Coastal Corridor. The corridor is defined by socio-political and biophysical boundaries; diverse coastal and marine ecosystems including wetlands, hypersaline marshes, mudflats, riparian systems, rocky reefs and sandy bottoms [52]. The coupled socio-ecological system supports the livelihoods of most of the coastal inhabitants of the Coastal Corridor. Artisanal fishers in the corridor target about 75 species in single and multispecific fisheries; sustainable fisheries production is hampered by coastal development, overfishing, unregulated fishing, insufficient enforcement of fisheries regulations, and the lack of information and tools for effective management [20,^48^,53].

**Table 1:** Target species selected as priority species in the CMSP process.

Common name	Spanish name	Species
Brown crab	Jaiba café	*Callinectes bellicosus*
Black murex snail	Caracol chino negro	*Hexaplex nigritus*
Pink murex snail	Caracol chino rosa	*Hexaplex erythrostomus*
Flatfish	Lenguado	Families Paralychtyidae and Pleuronectidae
Guitarfish	Guitarra	*Pseudobatos productus*
Pacific angel shark	Angelito	*Squatina californica*
Banded guitarfish	Cholo	*Zapteryx exasperata*
Brown smooth-hound shark	Tripa	*Mustelus henlei*
Gulf coney	Baqueta	*Hyporthodus acanthistius*
Gold-spotted sand bass	Extranjero / Cabrilla extranjera	*Paralabrax auroguttatus*
Gulf croaker	Chano norteño	*Micropogonias megalops*

## Method details

### Spatial prioritization methodology

To generate spatially explicit fisheries management tools, spatial prioritization was used software zonation [41]; (http://cbig.it.helsinki.fi/software/zonation). This software uses an algorithm that initially assumes that the entire landscape is protected and progressively identifies and removes cells that cause a lower marginal loss in the “value” of the landscape; removing these cells leaves higher value cells that are more relevant for spatial management given the priorities specified at the start of the analysis [39,40]. The algorithm defines the marginal loss, which allows specifying weights for each characteristic and connectivity between them; zonation can implement different types of marginal loss, including additive value [5].

The definition of marginal loss for core areas, used the following rules: 1) when considering two identical sites, the one with the lowest occurrence of the most important characteristics was removed first; 2) when considering two identical sites, the one with the occurrence of the characteristics with lowest weight was removed first; 3) assuming two sites with identical occurrence of two different characteristics, the one that has lost most of its distribution throughout was retained; and 4) out of two identical sites, the one with highest cost (based on weights provided for each feature) was removed first. The core area algorithm was used to guarantee the retention of high-quality areas for all species, including those areas with few species

Mathematically, the marginal loss of the area nucleus is defined by Eq. (1):

(1) $$\partial i=\max\frac{Q_{ij}\left(S\right)\omega_{j}}{C_{i}}=\frac{P_{ij}\omega_{j}}{C_{i}*\sum_{\kappa \in S}P_{kj}}$$

Where W _j_ is the weight of feature j, P _kj_ is the level of occurrence of feature j at site i, and Ci is the cost of adding cell i to the reserve network. The critical part of the equation is Q _ij_ (S), the proportion of the distribution of remaining species j located in cell i in the group of remaining cells, S. When a section of the feature distribution is removed, the proportion located in the remaining cells goes up. In this way, zonation tries to maintain high quality core areas for all characteristics until the end of cell removal, even if the characteristics are initially common and widely distributed.

zonation was used to generate potential sites for Locally Managed Marine Areas and Fisheries Refuges. The statistical program R (R Development Core Team 2012) was used for all analyses.

### Locally managed marine areas

Rights-based management areas were derived for each Corridor community for priority benthic and demersal species including brown crab, black murex snail, pink murex snail, flatfish, guitarfish, angelfish, and banded guitarfish (cholo). To determine the Locally Managed Marine Areas, several criteria were considered in the spatial prioritization models (Table 2). In general, models used the following data layers: (1) spatial boundaries for existing management instruments; (2) fishing intensity based on a standardization of fishing effort, derived from data obtained through various processes (interviews, fishing logs, and participatory mapping); (3) fishing areas for each community and species; (4) species distribution models, which correlate occurrence data and environmental variables, and represent the probability of finding a species in a given area; and (5) conflict areas identified between fishing sectors and others.

**Table 2:** Criteria used to assign priorities for the development of spatial prioritization models to select Locally Managed Marine Areas, ordered from highest to lowest importance for cell removal.

Preliminary criteria	Final criteria	Rationale (final criteria)
1. Areas allowed according to existing management instruments	1. Areas allowed according to existing management instruments	Removes areas as required by law
2. Higher fishing intensity	2. Management areas selected by the Intercommunity Fisher Group (IFG) (November 2015)	Removes the main fishing areas preferred by fishers
3. Higher likelihood of finding a species	3. Lower overlap with selected management areas for other communities	Minimizes conflict between communities
4. Lower overlap between communities’ fishing areas (for the same species)	4. Lower conflict with other fisheries and uses.	Minimizes conflict between fisheries
5. Lower conflicts with other fisheries and uses.	5. Higher likelihood of finding a species and fishing areas.	Eliminates areas with low abundance of target species
	6. Avoid rocky reefs.	Avoids reefs used for spawning and refuge
	7. Lower probability of incidental catch	Avoids areas with higher biodiversity

Table 1S (Supplementary information) gives more details regarding the data layers developed for this project, including layers for management boundaries. To determine areas of fishing importance and fishing intensity in the corridor, we used a database with georeferenced sites of fishing zones that include data taken from 2010 to 2012 for five communities in the corridor (excluding Puerto Peñasco). During this period, CEDO Intercultural tracked fishing locations for five target species for these five communities. Named fishing sites were georeferenced with a global positioning system. These data were combined with data collected in 2005 through interviews as part of the PANGAS project, where fishers were asked to map their fishing zones by species and resulting areas were then validated in workshops [42,43]. Both the geo-referenced sites and the fishing use polygons were converted into a grid. Fisheries importance was calculated as the sum of fishing events in each cell, normalized by the number of events by species and community. Fishing conflict zones, reproduction and breeding areas, and bycatch areas were delineated during participatory workshops.

**Fig. 2:**
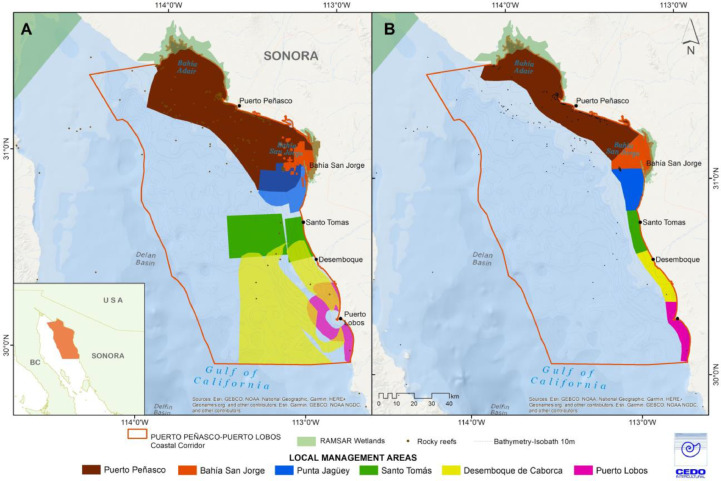
Proposed Locally Managed Marine areas based on spatial prioritization models. (A) for initial model runs, (B) after incorporating additional criteria and stakeholder input.

Five percent of the best zones chosen by zonation were selected and used to generate maps by species and coastal community with 1 km² grids, showing a gradation in color indicating the best areas (Fig. 2A). A second iteration of the spatial prioritization was run of models to incorporate community input: avoid rocky areas, include areas for spawning, nursery and juvenile aggregations, and areas of low abundance based on resource users’ traditional knowledge. The resulting area polygons were then presented to communities and modified manually, following agreements made between fishers from different coastal communities (Fig. 2B).

**Fig. 3:**
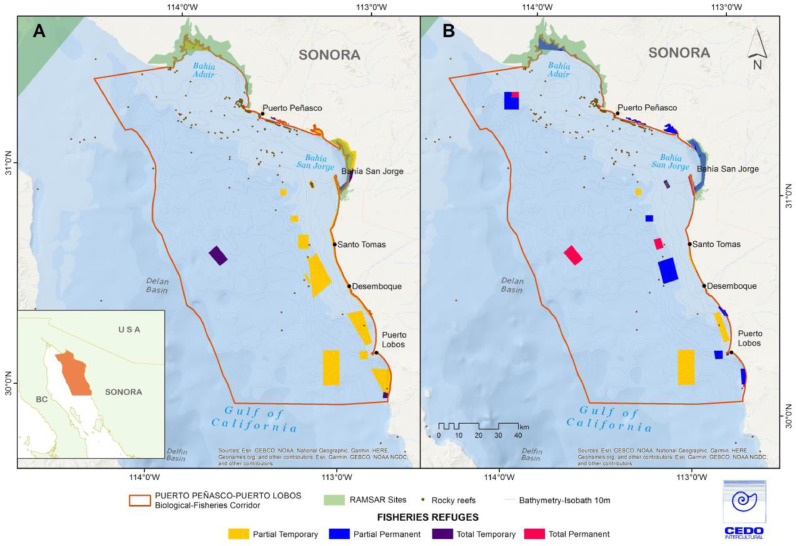
Proposed Fisheries Refuges derived from spatial prioritization models: (A) initial model runs, (B) final proposal, after incorporating additional criteria and stakeholder input. Refuge colors are based on the protection category as defined in existing fisheries regulations.

#### Fishery refuges

Using ZONATION models, we also identified potential global and species-specific Fishery refuge sites for all 11 priority target species (Table 1). Potential areas for establishment of Fishery refuges were selected using the four refuge categories described in existing fishery regulations that characterize types of gear restrictions and the time period for restrictions (Table 2S), and using the criteria described below in Table 3. In general, models of Fishery refuges considered the following data layers: (1) spatial data layers that delimit existing management areas in legal instruments; (2) fishing intensity models that standardized fishing effort derived from data from interviews, fishing logs, and participatory mapping; (3) the biodiversity index, based on occurrence records of species in the area; (4) species distribution models, which correlate occurrence data and environmental variables, and represent the probability of finding a species in a given area; (5) Fishery refuges proposed in fisher workshops where rights-based management areas were reviewed; (6) distribution of key habitats, rocky areas, and coastal wetlands; and (7) reproduction and breeding areas, identified from traditional ecological knowledge. The Biodiversity index was derived from the species richness model generated by interpolating occurrence records for unique species drawn from open-access databases as well as from primary literature in the Gulf of California [46]. Other data layers are explained under Locally Managed Marine Areas, above.

**Table 3:** Criteria used to generate spatial prioritization models for Fishery refuges. Ordered from highest to lowest importance for cell removal.

Global Fishery refuges	Rationale (final models)	Fishery refuges by species	Rationale (final models)
1. High biodiversity	Avoids areas with higher biodiversity of commercial species	1. Fishery refuges proposed in an initial exercise by the IFG	Favors refuges preferred by fishers
2. Areas protected by other legal instruments	Remove areas as required by law	2. Nursery and reproductive areas	Avoids reefs used for spawning and refuge
3. Fishery refuges proposed in an initial exercise by the IFG	Favors refuges preferred by fishers	3. Areas with high rates of incidental fishing in gillnets and longlines.	Avoids areas with higher species abundance
4. Key habitats	Avoids areas used for feeding and refuge	4. Highest probability of commercial target species distribution.	Avoids fishing areas with highest abundance
5. Nursery and reproductive areas	Avoids reefs used for spawning and refuge	5. Low fishing intensity	Favors areas fished infrequently
6. Areas with high rates of incidental fishing	Avoids areas with higher species abundance	6. Key habitats	Avoids areas used for feeding and refuge
7. Highest probability of commercial target species distribution.	Selects areas where species of interest are found	7. High biodiversity	Avoids areas with higher biodiversity of commercial species
8. Low fishing intensity	Favors areas fished infrequently		

These data were incorporated into zonation in conjunction with the specific criteria defined for this management tool with input from experts. Five percent of the best zones chosen through the spatial prioritization model were selected and used to generate maps (Fig. 2A). In general, in order to minimize the socioeconomic costs of implementation, stakeholders eliminated areas that were considered key to fishery livelihoods and made suggestions to modify the shapes of other areas. The areas were then modified as indicated and reviewed to ensure that they met the basic biophysical principles for design of a marine reserve network in the Gulf of California as outlined in [47], as follows: (1) habitat representation, with 10 to 30% of representative habitats present (rocky reefs, wetlands, rhodolith beds, mangroves and sandy and muddy bottoms; (2) risk dispersion, include at least 3 replicates of habitats per bioregion to diminish risks of hurricanes and large-scale disturbances; (3) protection for critical areas in the life history of priority species (larvae, juveniles and adults) with consideration for ontogenetic habitat shifts, including key habitat for species reproduction (estuaries and mangroves), reproductive aggregations and unique sites with extraordinary endemism, productivity and biodiversity; (4) maintain connectivity between refuges, considering that the home range movements of adults from focal species of fish depends on their maximum total length (e.g. species ≤ 167 cm requiere refuges at least 10 km long, or 100 km^2^). For species with planktonic larvae the location of sites that act as main larval sources depends on the spawning season, as oceanic currents that transport larvae reverse directions seasonally, and it also considers average larval dispersal distances (50 to 200 km, for planktonic larval durations 2–8 weeks, respectively); (5) considers that the full recovery of each species depends on its trophic level (e.g. ≥ 10 years for herbivorous and planktivorous fishes and ≥ 25 years for carnivorous and piscivorous fishes), and allows for sufficient recovery time, preferably permanent establishment, needed for conservation and fisheries recovery; (6) establish refugia in sites that are resilient to climate change and changes in ocean chemistry, and consider the effects of climate change on the distribution, development, growth and reproduction of species and on ecosystem function and dynamics; and (7) minimize or avoid local threats, avoid areas already impacted and areas that would increase vulnerability of local communities.

Given the 200 km total length of the corridor, this exercise was approached by subdividing the corridor into four 50 km sections, corresponding to the minimum distance necessary to assure adequate larval dispersal for corridor target species with short planktonic larval duration (2–3 weeks, e.g. black and pink murex snails). The proposed network had more Fishery refuges located upstream that could act as larval sources, given the northward prevailing oceanic current during the spring-summer spawning period of most commercial species with planktonic larvae. Refuges were also located in areas that show high levels of local larval retention and that could self-replenish, such as Bahia San Jorge [53]. We reviewed the refuges proposed for each subregion to assure the presence of refuges for all habitats used during ontogenetic habitat shifts (rocky, sandy/muddy, rhodolith beds, mangroves, and wetlands) within each subdivision and then checked for duplicity of habitats within a subdivision, with a minimum of two. We did the same to assure that there was replication of refuges for each priority species, both within and between sub-regions. We checked that the proposed refuges included a range of sizes that was adequate according to the home-range movements of the priority species of fishes (gulf coney: 178.7 km^2^, gold-spotted sand bass: 6.7 km^2^, gulf croaker: 1.08 km^2^, flatfish: 3.92 km^2^, brown smooth-hound shark: 19.80 km^2^, pacific angel shark: 71.7 km^2^, guitarfish: 106.7 km^2^, banded guitarfish: 16.89 km^2^). We also verified that the refuges had compact shapes that minimize edge effects that might reduce the quality of habitat, except when refuges were located within whole ecological units (e.g. estuaries).

Ways to simplify the network were sought to facilitate ease of implementation. The type of refuge was sometimes altered to align with others in the area to reduce the number of different rules that would have to be implemented in a community; some refuges were combined, and the shape of others was modified to simplify establishment, all supported by community input. The communities were returned to obtain final approval which was granted and documented in minutes and in letters of agreement obtained in January and February 2018.

#### Quota prioritization

Fishing quotas establish the quantity of a resource that can be extracted during the fishing season, helping to maximize resource use under the principle of maximum sustainable yield [12]. Quotas thus establish controls on fishing mortality based on their biological availability, provide certainty to the fishing sector about the volume of resources they can extract during the fishing season, and help maximize economic benefits under the principle of maximum sustainable yield. Quotas must be set in compliance with other existing restrictions (for example, closures), and can be set in conjunction with limitations on fishing gear, minimum sizes, and retention of reproductive individuals. Once a quota has been established, regular stock analysis must be carried out (eg. annually) and larval recruitment should be assessed to determine the biological status of the population. In Mexico, there is no established methodology for quota estimation; quotas can be determined based on density and abundance data (length-weight relationships), or using empirical studies or models that consider uncertainty in biomass estimation. The final output is a quota based on the estimated escapement. Under Mexican legislation, quota determination is the responsibility of the Mexican Fisheries Agency. Using available catch and biological data generated from the project, we conducted a preliminary analysis to prioritize the use of catch quotas for the selected target species as a way of providing initial information to the responsible government parties to forward quota implementation.

The life-cycle characteristics of 11 priority species in the Coastal Corridor were evaluated to identify good candidate species for quota implementation (Table 4). Fisheries and species’ life cycle characteristics can potentially limit or favor establishment of catch quotas, as follows: (1) In a multi-specific fishery, for example, the quota estimation and effectiveness of application could be affected, since different species have different reproductive and nursery areas, different migratory patterns, age at first maturity, reproductive output and recovery time, etc.; (2) If the species is sedentary, it facilitates the establishment of management areas and the determination of biomass, favoring quotas; (3) If the species is migratory, quotas might be difficult to apply across the entire extent of distribution; and (4) Maximum age and growth rate, may impact whether the benefits of quota establishment will be realized in the same time scale as management implementation, especially species with long life histories and slow growth rates. Based on these considerations, we determined that flatfish and gulf coney (*Hyporthodus acanthistius*) would be excluded from the quota prioritization exercise, because flatfish are a multi specific fishery, while gulf coney has a long life history and a late age of first maturity, both factors that are not recommended for the use of quotas.

**Table 4:** Characteristics of the life cycle of the corridor species, used to determine the feasibility of quotas. (-) indicates no data available.

Characteristics	Pink mmurex snail	Black murex snail	Brown crab	Flatfish	Species Pacificangel shark	Banded guitarfish	Guitarfish	Gulf Coney	Gold- spotted sand bass	Gulf croaker	Brown smooth-hound shark
Sedentary species	X	X	–	–	–	–	–	–	–	–	–
Migratory species							Coastal Zone				Coastal Zone
Maximum age	4–5	4–5	8	-	-	-	11–16	28	16–24	10	9–14
Age first maturity	2–3	–	1	-	-	–	5–7	7	4	–	3–4
Multispecies fisheries	–	–	–	X	–	–	–	–	–	–	–

A constraint analysis was performed to prioritize quota implementation for key target species. This analysis is a rapid and semi-quantitative risk assessment of a resource based on its biological productivity and susceptibility to fishing. To do the analysis three tools were applied from the Fishing Toolbox of the Environmental Defense Fund-FISHE (http: // fishe.edf.org /): (1) the Productivity and Susceptibility Analysis (PSA) model; (2) An initial stock assessment; and (3) SEASALT: a tool to analyze management attributes. Each of these steps is summarized below and detailed in Table 5.

**Table 5:** Criteria used for SEASALT, management-based attribute analysis.

Attribute	Criteria for evaluation
Secure	Tenure length of fishing rights Renewal of fishing rights Ability to defend rights legally
Exclusive	Rights are clearly defined by the allocation of quotas Penalties for violation of privilege by third parties Effect of new entrants on existing fees
All sources	Assignment of arrivals and discards by catch Controls on fishing mortality incorporate other fleets or sport / recreational users who fish the same stocks
Scaled	Population is managed by a single well-coordinated and accountable unit Political, cultural, social and economic differences between the fishing groups within the program are integrated into the design
Accountable	Participatory resource management There are mechanisms to enforce community regulations, standards and / or agreements Monitoring systems are up and running
Limited	Best available science is used to limit fishing mortality
Transferability	Not applicable

### Productivity and susceptibility analysis (PSA) model

The productivity model and susceptibility analysis (PSA) offers an assessment of the vulnerability of the populations subject to fishing pressure using basic biological and fishery information. This method was developed for fisheries with limited data and is used to evaluate populations where no stock analysis is available. They are only used as characteristic parameters to describe the life cycle with age at first maturity, maximum age, fecundity, natural mortality and behavior (Table 3S). These data are available in data bases such as FishBase, publications, or directly as traditional knowledge of fishermen. No catch records, effort estimation or independent monitoring of the fishery are required for this analysis. The productivity, or potential rate of population growth and the susceptibility of the stock to the fishing pressure are rated on a scale of 1 to 3. The final grade for productivity and susceptibility is calculated according to Eq. (2); where w is a weight (0 to 4) assigned to each attribute.

This weight represents the utility of the parameter to determine the vulnerability of the stock; in general; it is given the value of 2 unless there are specific reasons to the contrary. A value of 0 removes the attribute from the analysis. The final vulnerability score is the result of the individual attributes, estimated according to Eq. (3), where p is productivity and s is susceptibility; so that the influence of any attribute is relatively small. The data we used for each species to apply the PSA is found in Table 4S

(2) $$\frac{\sum_{i=1}^{n}w}{\sum_{i=1}^{n}a*w}$$

(3) $$\sqrt{{\left(p-3\right)}^{2}\;+\;{\left(s-1\right)}^{2}}$$

The PSA takes into account the quality of the data used, ranging from the complete absence of data to quality data obtained through formal population assessments; a score of 1 to 5 is assigned indicating higher to lower quality data (Table 5S shows the data quality scores used as part of the analysis). This score allows us to incorporate uncertainty into the vulnerability analysis. To carry out the PSA, we used the tool developed as part of the NOAA Fisheries Toolbox, available at http://nft.nefsc.noaa.gov/index.html.

#### Stock assessment

Three Froese Sustainability Indicators [23] were used to estimate the status of the stock, these indicators are:

1. Percentage of mature fish in the catch. Mature individuals are defined as those which have had a chance to spawn at least once; this is determined by the length at first maturity and converted into the length at which 90–100% of the fish of a given species are mature. The target is to let all (100%) fish spawn at least once before being caught (i.e., zero catch of juveniles) to rebuild and maintain healthy spawning stocks, 90% is a reasonable target.
2. Percentage of fish caught at the optimum length for harvest (minimizing any adverse impacts of fishing). Optimum length is where the number of fish in a given unfished year-class, multiplied by their mean individual weight is highest, resulting in maximum yield. The target is to have all harvested fish (100%) be within +/- 10% of optimum length
3. Percentage of megaspawners in the catch. Megaspawners are older females that tend to produce more, larger, and qualitatively superior eggs [54]. If the catch reflects the age structure of the stock, 30–40% of megaspawners in the catch would likely represent a healthy population, with 20% being a lower limit. The target is to harvest no (0%) mega-spawners.

The data used to calculate these indicators included: (a) the size frequency of the catch; (b) the size at first capture; (c) the length of first maturity; and (d) the maximum theoretical length. Initially, a length-frequency analysis was carried out and the indicators were estimated using the FishBase estimation tool (http://www.fishbase.org). We did not estimate indicators for pacific angel shark, since there was no information on the size structure of the catch in the Northern Gulf of California. Since a sample number of 500–1000 specimens is recommended for this analysis, we also did not estimate the indicators for banded guitarfish, with only 28 records. The analysis for banded guitarfish, gold spotted sand bass, and gulf croaker have a higher degree of uncertainty since there are <300 records for these species. Table 6S and Fig. 1S show the size frequency analysis and the parameters used to calculate the Froese Sustainability Indicators. These data were obtained from CEDO's community catch monitoring program, other biological monitoring efforts [48], and FishBase [24], using its *Length-Frequency Analysis Wizard.* Table 7S shows how the final Froese Sustainability Indicators were estimated.

**Table 6:** Scenarios tested using the Atlantis Ecosystem Model for the Northern Gulf of California. Scenarios represented combinations of management instruments simulated as direct reductions in fishing mortality or partial or total closures of model polygons.

Scenarios	Effect	Levels	Species with modelled effects
1–3. Reduction of fishing mortality	Fishing mortality by fleet and species	−10% −1% Multispecific groups in Atlantis	1. Only species evaluated for quotas 2. All priority fishery species 3. Species extracted in corridor by outside communities 4. Species with existing management processes and plans
4. Fishery refuges	Space restrictions	Restrictions on proposed refuges	1. Species extracted by affected fleets
5–8. Reduction Fishing mortality + Fishery refuges	Fishing mortality by fleet and species Space restrictions	−10% −1% Multispecific groups in Atlantis Restrictions on proposed refuges	1. Species evaluated for quotas 2. Priority fishery species 3. Species extracted in corridor by communities outside 4. Species with management committees

#### Management attributes

The **SEASALT** tool developed by Environmental Defense Fund was used [8], which employs expert knowledge to evaluate fishery management attributes (Table 5). This tool assesses the attributes needed for a successful catch share program: (1) Secure - establishment can be sufficiently long to realize future benefits; (2) Exclusive - rights adjudicated to a group or individual are recognized and defendable by law; (3) All sources - all sources of fishing mortality are accounted for and don't exceed the catch limit. (4) Scaled - management units are set at appropriate levels; (5) Accountable - participants can stay within their allocation of overall catch; (6) Limited - the catch limits are set within appropriate biological levels. A final attribute evaluates Transferability, but this is not applicable because under Mexican law quotas are not transferable. We carried out interviews with community members and stakeholders to qualitatively evaluate the state of each attribute for each species and the perceived tendency. The qualitative state was evaluated in a scale from 5-0, where (5) superior, (4) good, (3) average, (2) poor, (1) critical, and (0) indeterminate. The perceived tendency was also evaluated on a 5–0 scale, where (5) there are actions that have been taken or are planned in the future that could strongly and positively affect the indicator, (4) there are actions that have been taken or are planned in the future that could positively affect the indicator, (3) there are no actions that have been taken or are planned in the future, (2) there are actions that have been taken or are planned in the future that could negatively affect the indicator, (1) there are actions that have been taken or are planned in the future that could strongly and negatively affect the indicator, (0) there is insufficient information to determine the future development of the indicator. Table 8S has the detailed score determination for each attribute in Table 5.

### Regularization of permits

The fishing permit represents the basic legal mechanism, whereby an individual or a fishing institution (cooperative) obtains the right to fish. The permit designates the area where fishers are allowed to fish, but also may stipulate specific restrictions such as Fisheries refuges and catch quotas. Concessions represent an alternative to the permit, which is a longer term contract for exclusive use of a resource or area.

**Fig. 4:**
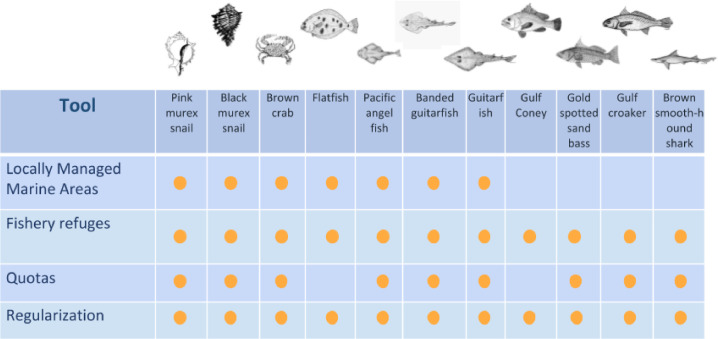
Management tools applied to each priority species.

As illustrated in Fig. 4 regularization of permits is considered an integral part of the spatial management framework for this CMSP process. By aligning fishing permits to the actual documented fishing effort, whether regular or irregular, and clarifying the boundaries of these permits, the rights of stakeholders, especially fishermen, involved in this process are strengthened, and with this, stewardship. The trade-off analysis considers the fishing mortality associated with the actual fishing effort within the corridor, but tests scenarios where this mortality increases when outsiders have access to the region.

### Trade-off analysis

**Fig. 5:**
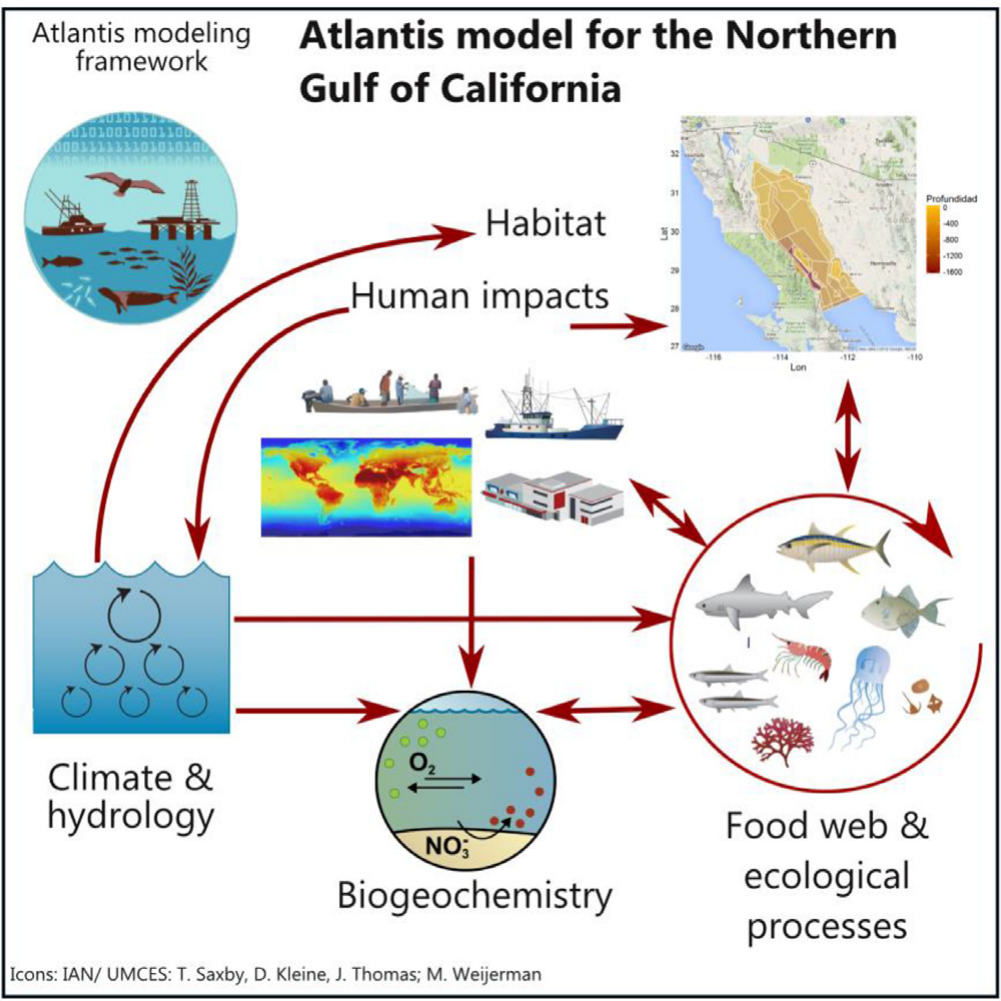
Structure of the Atlantis ecosystem model for the Northern Gulf of California.

A trade-off analysis was performed to evaluate the application of the four management tools selected for each species, including Locally Managed Marine Areas, Fishery Refuges, quotas (Fig. 4), using an Atlantis ecosystem model for the Northern Gulf of California [1]; Fig. 5). Atlantis is an end-to-end ecosystem modeling approach that integrates physical, chemical, ecological, and fisheries dynamics in a three-dimensional, spatially-explicit domain that uses an irregular polygon structure to represent important bioregional features [25]. The modeling platform summarizes biological components as functional groups aggregated by trophic, life history, or niche similarities. Further information on Atlantis can be found in the User's Guide [6], the Atlantis Wiki (https://research.csiro.au/atlantis/home/links/), and recently published Atlantis applications [29,^49^,50]. The Atlantis model for the Northern Gulf of California, which includes the Coastal Corridor, extends over 57,800 km^2^, represents ecosystem structure and function in 2008, current fishing effort, and provides a detailed representation of the Northern Gulf's oceanography, historical fishing patterns, migration and movement of key species, and variability in diet compositions [2,3].

Atlantis operates in a three-dimensional structure that mimics the depth profile and geography of the study area, including Marine Protected Areas, fishing activities by fleet, stock trends, indicators of the structure and function of the ecosystem and human sectors. The model was built to explore both ecosystem-based fisheries management questions and ecological hypotheses. To date the model has been used to test the effect of fishery regulations [2,2] and conservation policies [44,45] on the marine ecosystem of the Northern Gulf.

We modeled scenarios (Table 5) composed of specific combinations of the above management tools, simulated as reductions on fishing mortality either on specific functional groups or across model polygons (Figure 2S). The scenarios represent the effects of the Locally Managed Marine Areas, Fisheries Refuges and fishing quotas through a reduction in fishing mortality with respect to that represented in the model. This reduction was applied to the species for which spatial management areas were developed, species that were selected for prioritization of fishing quotas, species that were identified by stakeholders as species extracted by communities outside the corridor and other key species.

The base scenario included spatial restrictions for Marine Protected Areas (Figure 3S) and other existing management instruments (Table 9S) in the Northern Gulf. The simulations were projected for 26 years from 2008 to 2033. Direct reductions in fishing mortality were implemented in 2018 and maintained during the simulation. The Fisheries refuges, implying partial or complete closures of model polygons for specific gears according to the restrictions in each refuge, were implemented in 2019 and maintained during the duration of the simulation, representing three consecutive 5-year ‘renewal’ periods. We analyzed outputs at 5, 10, and 15 years after the application of the fishery refuges for model polygons within the Coastal Corridor.

The trade-off analysis was used to evaluate benefits between alternative scenarios using 10 ecosystem indicators that represent aspects of ecosystem structure, resilience, and fishing. These indicators used inputs that resulted from the simulations of the Atlantis model including biomass, catch, number, and size of fish. The indicators analyzed were: (1) ecosystem structure and resilience, including: (a) biomass, (b) biodiversity, (c) proportion of juvenile fish, (d) trophic level and (e) ratio of pelagic to demersal fish; and (2) fishery health as measured by: (a) trophic level of catch, (b) maximum size in catch, (c) value of catch, (d) fish catch, and (e) biomass of commercial species. To calculate biodiversity, we used the Q-90 statistic [4], which represents the slope of the cumulative species abundance curve and reflects both species evenness and richness based on the 54 major vertebrate and invertebrate functional groups in the model. Fish body size was calculated based on reserve nitrogen, which represents weight-at-age of muscle, fat, reproductive parts, and other soft tissue. To assess trade-offs between scenarios and the impact of management tools on indicators, we presented results using radar graphs that visualize indicators across scenarios in a normalized scale. The simulation results generally indicate the degree to which the reduction in mortality and the application of refuges can benefit the ecosystem. The scenarios that combine refuges with mortality reduction show greater benefits than any of those factors alone. Figure 4S shows an example of how radar graphs were used to show alternative benefits between indicators and scenarios.

Supplementary material *and/or* Additional information:

## Declaration of Competing Interest

X The authors declare that they have no known competing financial interests or personal relationships that could have appeared to influence the work reported in this paper.

## Appendix Supplementary materials

Image, application 1
