# Supplementary material for: Fisheries management tools to support coastal and marine spatial planning: A case study from the Northern Gulf of California, Mexico
Source: MethodsX. 2020 Oct 16;7:101108. doi: 10.1016/j.mex.2020.101108 (PMC7596294; doi:10.1016/j.mex.2020.101108)

Supplementary Information

Fisheries management tools to support Coastal and Marine Spatial Planning: A case study from the Northern Gulf of California, Mexico

Hem Nalini Morzaria-Luna[1,2]*(*a*), Peggy Turk-Boyer[1,3](*a*), José Manuel Dorantes Hernández[4], Elia Polanco-Mizquez[1], Caroline Downton-Hoffmann[1], Gabriela Cruz-Piñón[1,4], Tonatiuh Carrillo-Lammens[1], Rene Loaiza-Villanueva[1], Paloma Valdivia-Jiménez[1], Angeles Sánchez-Cruz[1], Valeria Peña-Mendoza[1], Ariadna Montserrat López-Ortiz[4], Volker Koch[5,6] Leonardo Vázquez-Vera[7], José Alfredo Arreola-Lizárraga[8], Imelda G. Amador-Castro[9], Alvin N. Suárez Castillo[9], Adrian Munguia-Vega[9,10]

*Corresponding author (email: hemnalini@cedointercultural.org). Ph: 206-384-2466
ORCID: 0000-0001-5265-1049

(*a*) Both authors contributed equally to this paper.

1. CEDO Intercultural. Apartado Postal #53. Puerto Peñasco, Sonora, México C.P. 83550. / P.O. Box 44208, Tucson, AZ 85733, USA. PTB, peggy@cedointercultural.org, TCL, tonatiuh.carrillo@gmail.com; EPM, elia@cedointercultural.org; CDH, caroline@cedointercultural.org; RLV, rene@cedointercultural.org; PVJ, paloma@cedo.org; ASC, angeles@cedointercultural.org; VPM, vpenamendoza@gmail.com
2. Visiting Researcher, Northwest Fisheries Science Center, NOAA. 2725 Montlake Blvd. Seattle WA 98112, USA. HemNalini.MorzariaLuna@noaa.gov
3. Director Emeritus. CEDO Intercultural. P.O. Box 44208, Tucson, AZ 85733, USA.
4. Departamento de Ciencias Marinas y Costeras. Universidad Autónoma de Baja California Sur. Carretera al Sur Km 5.5, Apartado Postal 19-B, C.P. 23080. La Paz, Baja California Sur. JMDH, dorantessp@gmail.com, GCP, gacruz@uabcs.mx; AMLO, ariadnaamlo@gmail.com
5. Deutsche Gesellschaft für Internationale Zusammenarbeit (GIZ) GmbH, P.O. Box 5180, 65726 Eschborn, Germany. vokoch68@gmail.com
6. Departamento de Ecología Marina, Centro de Investigación Científica y Educación Superior de Ensenada, Ensenada, Baja California, México.

7. The Nature Conservancy. La Paz, Baja California Sur. 23000. leonardo.vazquez@tnc.org
8. Centro de Investigaciones Biológicas del Noroeste, S.C. Campus Guaymas, Km 2.3 carr. a Las Tinajas predio El Tular s/n , Guaymas, Sonora, México, CP. 85454. aarreola04@cibnor.mx
9. Comunidad y Biodiversidad, A.C. Isla del Peruano 215, Lomas de Miramar, Guaymas,Sonora, CP. 85448. ANSC, sargassumgc@gmail.com; IGAC, iamador@cobi.org.mx
10. Desert Laboratory on Tumamoc Hill , University of Arizona, Tucson, AZ, 85721, USA. airdrian@email.arizona.edu

Table 1S. Summary of spatial layers used in ZONATION spatial prioritization models

Space layer	Category	Source
Biodiversity index	Ecological feature	Number of species weighted by sampling effort, Morzaria-Luna et al.,2018.
Alto Golfo and Colorado River Delta Biosphere Reserve	Management area	CONANP (2007). Designated area and zoning and sub-zoning of use http://sig.conanp.gob.mx/website/pags/ig/
Species distribution models	Ecological feature	Potential distribution of species based on presence records that provide the probability of occurrence of species. COBI / UABCS (unpublished information)
Gillnet exclusion zone, falsework and / or longlines for protection “vaquita marina”	Fisheries restrictions	DOF (2015)
Responsible fishing for sharks and rays	Fisheries restrictions	NOM-029-PESC-2006 (DOF 2006a)
Rocky reef	Habitat	Submarine transect-based mapping, knowledge of artisanal, recreational and industrial shrimp fishermen, 412 records. (CEDO Intercultural 2003)
Conflicts between communities	Human use	Participatory mapping in workshops for conflict identification, October 2015
Breeding and breeding areas	Ecological feature	Breeding and breeding areas indicated in participatory workshops with communities (April-May 2016).
Incidental fishing	Human use	High bycatch areas identified in participatory workshops with fishermen, April-May 2016
RAMSAR wetlands	Management area	CONANP (2009) http://sig.conanp.gob.mx/website/pags ig/
Fishing importance	Human use	Fishing intensity by species derived from a normalized index from CEDO, unpublished data from: Beach landings and interviews with fishermen 2010-2012. 20,758 fishing.trips Munguía-Vega et al. (2015). Fishing logs; 4882 spatial records of fishing areas generated through participatory mapping by fishers(Moreno-Báez et al. (2012).

Table 2S. Characteristics of Fishery refuges described in the Mexican regulation NOM-049-SAG/PESC-2014. Four types of refuges are described by Mexican law based on the extent of gear restrictions and the period these restrictions are implemented.

	Gear restriction	
**Duration**	**Total**	**Partial**
**Temporary**	No fishing activities permitted during a defined period. Catch and release sport fishing allowed.	Sport fishing, commercial, research, and subsistence fishing are only allowed with highly selective gears during a defined period.
**Permanent**	No fishing activities permitted	Sport fishing, commercial, research, and subsistence fishing are only allowed with highly selective gears.

Table 3S. Productivity attributes and score categories (1-3) that are assigned as part of the Productivity and Susceptibility Analysis (PSA) model.

Productivity Attributes	Definitions	High (3)	Moderate (2)	Low (1)
r (population growth)	Intrinsic rate of population growth or maximum population growth that is expected from a population in natural conditions (eg without fishing) and that directly reflects the productivity of the stock.	>0.5	0.5-0.16 (middle point 0.10)	<0.16
Maximum age	The maximum age is a direct index of the natural mortality rate (M), low values of M are indirectly correlated with high maximum ages (Hoenig 1983).	<10 years	10-30 years (middle point 20)	>30 years
Maximum age	The maximum size is correlated with productivity. Large fish tend to have low levels of productivity (Roberts & Hawkins 1999), this relationship tends to degrade at high taxonomic levels.	<60 cm	60-150 cm (middle point 105)	>150 cm
Von Bertalanffy growth coefficient (*k*)	The Von Bertalanffy model measures the speed at which a fish reaches its maximum size. Long-lived fish in unproductive stocks tend to have low values of k (Froese & Binohlan 2000).	>0.25	0.15-0.25 (middle point 0.20)	<0.15
Mortality natural estimated (M)	The natural mortality rate directly reflects the productivity of the population, stocks with high rates require high production levels to maintain population levels.	>0.40	0.20-0.40 (punto medio 0.30)	<0.20
Measure fertility	Fecundity (the number of eggs produced by a female in a reproductive event or period), is measured at the age of first maturity.	>10e4 (100,000)	10e2-10e3 (1,000-10,000)	<10e2 (1,000)
Reproductive Strategy	The reproductive strategy of a stock provides an indication of the level of mortality that is expected of the progeny in their first stages of life.	0	Between 1 y 3	≥4
Pattern recruitment		Recruitment success very frequent (> 75% of classes per year are successful)	Moderately frequent recruiting success (between 10% and 75% of classes per year are successful)	Recruitment success rare (<10% of classes per year are successful)
Age of maturity	Age of maturity tends to relate positively with maximum age (tmax). Long-lived low-productivity stocks will have higher maturity ages compared to stocks with low longevity.	<2 years	2-4 years (middle point 3.0)	>4 years
Trophic level medium	The position of a stock in the fish community can be used to infer its productivity, the stocks that are in the trophic base are generally more productive than those at the tip.	<2.5	2.5-3.5 (middle point 3)	>3.5

Table 3S (continued). Productivity attributes and score categories (1-3) that are assigned as part of the Productivity and Susceptibility Analysis (PSA) model.

Susceptibility attributes	Definition	Low (1)	Moderate (2)	High (3)
Management strategy	The susceptibility of a stock to overfishing depends on the effectiveness of the fishery management procedures used to control the catch.	Target populations have catch limits and proactive liability measures; non-target populations are carefully monitored.	Target populations have catch limits and shared responsibility measures	Target populations do not have catch limits or management measures; non-target populations are not carefully monitored
Superposition of areas	Measurement of geographic overlap between the known distribution of a stock and the distribution of the fishery.	<25% of the population occurs in the fishing area	Between 25% and 50% of the population is in the fishing area	> 50% of the population is in the fishing area
Geographical concentration	Degree that a stock is concentrated in small areas	The population is distributed in> 50% of its total range	The population is distributed in 25% to 50% of its total range	The population is distributed in <25% of its total range
Vertical overlay	The position of a stock in the water column (i.e. demersal or pelagic) relative to the fishing gear	25% of the population is in the depth where it is caught	Between 25% and 50% of the population occurs in the depths where fishing takes place	> 50% of the population occurs in the depths where fishing takes place
Fishing rate Related with M	Only applies to stocks where estimates of the fishing mortality rate (F) and (M) are available.	<0.5	0.5-1.0	>1
Biomass reproducers (SSB) and other proxies	Analogous to the fishing mortality, the degree to which fishing has decreased the biomass of the stock relative to the unfished level; offers an indication of susceptibility	B is> 40% of B0 (or maximum observed from the time series of the biomass estimates)	B is between 25% and 40% of B0 (or maximum observed from the time series)	B is <25% of B0 (or maximum observed)
Seasonal migrations	Movement from or towards the fishing area (eg spawning or food migrations) that could affect the overlap between the stock and the fishery.	Seasonal migrations reduce their overlap with fishing	Seasonal migrations do not significantly affect the overlap with fishing	Seasonal migrations increase their overlap with fishing
Schools / Aggregation	Behavioral responses in individual fish and stock in response to fishing.	Behavioral responses reduce the probability of capture of fishing gear	Behavioral responses do not significantly affect the probability of catching the fishing gear	Behavioral responses increase the probability of capture of fishing gear
Morphology that affects the catch	The ability of the fishing gear to catch fish based on the morphological characteristics of the fish (eg body shape, spiny fins or soft rays).	The species shows low selectivity to fishing gear	The species shows moderate selectivity to fishing gear	The species shows high selectivity to fishing gear
Survival after capture and release	Survival of the fish 'catch and release' varies by species, region, type of equipment, including market conditions, and affects the susceptibility of stock.	Probability of survival> 67%	Probability of survival between 33% and 67%	Probability of survival <33%
Convenience value of the fishery	This attribute assumes that highly valued stocks are more susceptible to overfishing by commercial or	Fishing does not value or highly desire this population	Fishing values or want moderately this population	Fishing values or highly values this population

	recreational fishing due to the increase in effort.			
Impact of the fishery about habitats fish essentials (EFH) or habitats in general (not target species)	A fishery can have an indirect effect on a species through impacts on the habitat.	Adverse effects are absent, or are minimal or temporary	Adverse effects are greater than minimum or temporary but are mitigated	Adverse effects are greater than minimum or temporary and are not mitigated

Table 4S. Data used for the Susceptibility and Vulnerability Analysis. Attributes are shown for females (F) and males (M) when available. Weight and attributes are scored 0-4. Weighted attribute score = Weight * Attribute score. Weighted data quality score = Weight * data quality score.

Black murex snail

Productivity Attributes	Data	Source	Weight	Attribute score	Weighted attribute score	Data quality score	Weighted data quality score
R	NA		2	2	4	2	4
Maximum age	At least 8 years (CEDO 2012). 12 years (Valdez-Ornelas, 2011)	Cudney-Bueno & Rowell 2008 / Valdez-Ornelas 2011	2	3	6	2	4
Maximum size	180 mm (Cudney-Bueno y Rowell 2008) 190 mm (CEDO 2012). 175 mm (Biometrics CEDO 2010). 142 mm (Salazar Navarro et al. 2008). Linf 184 mm (Valdez-Ornelas 2011)	CEDO 2012, Biometrics CEDO 2010, Salazar Navarro et al. 2008, Valdez-Ornelas 2011, Cudney-Bueno & Rowell 2008	2	3	6	1	2
Growth coefficient von Bertalanffy (k)	0.31 K annual	Valdez-Ornelas 2011	2	3	6	2	4
Estimated natural mortality (M)	0.6 M (annual)	Valdez-Ornelas 2012	2	3	6	2	4
Measured fertility	3603±221 embryos / capsule (Cudney et al. 2008).1583 ±149 embryos / capsule (Góngora Gómez et al. 2011). 285 a 420 Capsulas/individuals; 500,000 a 1'500,000 larvae annual fertility (Ficha, 2012).	Cudney et al. 2008; Góngora-Gomez et al. 2011. CEDO 2012.	2	2	4	1	2
Reproductive strategy	Reproductive aggregations. "r" Strategy. Lay capsules on other shells of other species; one snail can deposit hundreds of eggs	CEDO 2012; SeaLifeBase / Prescott & Cudney-Bueno 2008	2	3	6	2	4
Pattern recruitment	Annual/summer	CEDO 2012	2	3	6	2	4
Age of maturity	2 to 3	Cudney-Bueno & Rowell 2008	2	2	4	2	4
Medium trophic level	2.98	Valdez-Ornelas 2011	2	2	4	2	4
Results final Productivity					2.6		

Table 4S (cont.)

Black murex snail							
**Susceptibility attributes**	**Data**	**Source**	**Weigh**	**Attribute score**	**Weighted attribute score**	**Data quality score**	**Weighted data quality score**
Management strategy	Fishing permits, exploitation rate of 40% of the biomass and minimum capture size of 90 mm. Recommends a fishing ban from May 1 to July 31.	Carta Nacional Pesquera (2012). CEDO (2012)	2	2	4	2	2
Superposition of Areas	The corridor area has the highest proportion of the stock biomass	Loaiza et al. 2012	2	3	6	2	1
Geographical concentration	Although it is found from the coast of California to Oaxaca, the fishery is formally done only in the Northern Gulf	Cudney, Turk-Boyer 1998; Ficha 2012	2	3	6	1	2
Vertical Superposition	Snails are typically found on the surface of the seabed during the months of warm water and are buried in the sediments during the months of cold water	Prescott and Cudney-Bueno 2008	2	3	6	2	2
Fishing fee related to M	Variable data by size. Attached table Valdez Ornelas 2011	Valdez Ornelas 2011	2	2	4	2	2
Breeder biomass (SSB) or other proxies	2,152 ton biomass required for recovery.	Valdez Ornelas 2011.	2	2	4	2	1
Seasonal migrations	It is dispersed in fine, thick sand and tepetate to feed outside the reproductive period between 5 and 15 m deep	Loaiza, et al. 2012	2	3	6	1	2
Schools/ Aggregations	It is added in areas of coarse sand-conchal and tepetates for reproduction	CEDO 2012	2	3	6	2	1
Morphology that affects the capture	None, only behavior		2	3	6	1	2
Survival after capture and release	No data		2	1	2	3	3
Convenience / value of the fishery	High fishery value for diving fisheries. Exploitable biomass 1,012 Ton ($10 MEX/kg beach price).	*Com. Pers. Alejandrino Madueño June 2016	2	1	2	2	2
Impact of the fishery on essential fish habitats (EFH) or habitats in general	The direct impact to the habitat from the fishery is unknown.	CEDO 2012	2	1	2	2	2
Final result Susceptibility					2.25		
Final result vulnerability			1.31				

Table 4S (cont.)

Pink murex snail							
**Productivity attributes**	**Data**	**Source**	**Weight**	**Attribute score**	**Weighted attribute score**	**Data quality score**	**Weighted data quality score**
r (population growth)	0.91	Baqueiro et al. 2003.	2	3	6	2	4
Maximum age	8 years	Based on Black murex, is the closest with field data	2	2	4	3	6
Maximum size	127 mm	CEDO Field data 2013	2	2	4	1	2
Growth coefficient von Bertalanffy (k)	0.65	Baqueiro et al. 2003.	2	3	6	2	4
Estimated natural mortality (M)	0.93	Baqueiro et al. 2003.	2	3	6	2	4
Measured fertility	3603 ± 221 embryos / capsule (Cudney, et al., 2008).1583 ± 149 embryos / capsule (Góngora Gomez et al., 2011). 285 to 420 Capsules / individuals; 500,000 to 1'500,000 annual fertility larvae (CEDO, 2012).	Based on Black murex, is the closest with field data	2		0	3	6
Reproductive strategy	Reproductive aggregations. "R"Strategy	CEDO 2012	2	3	6	2	4
Recruitment pattern	Annual/summer	CEDO 2012	2	2	4	2	4
Age of maturity	2 a 3	CEDO 2012	2	2	4	2	4
Average trophic level	2.1	CDH 2015	2	3	6	2	4
Final result productivity					2.3		

Table 4S (cont.)

Pink murex snail							
**Susceptibility attributes**	**Data**	**Source**	**Weight**	**Attribute score**	**Weighted attribute score**	**Data quality score**	**Weighted data quality score**
Management Strategy	Fishing permits, exploitation rate of 40% of the biomass and minimum capture size of 90 mm. Recommends a closure from May 1 to July 31	Carta Nacional Pesquera 2012 CEDO 2012	2	2	4	2	4
Superposition of areas	The corridor area has the highest proportion of the stock biomass	Loaiza et al. 2012	2	3	6	2	4
Geographical concentration	It inhabits from the Gulf of California to Peru	Fischer 1995	2	2	4	2	4
Vertical Superposition	Shallow coastal zone up to 50 m depth	Carta Estatal Pesquera Baja California 2016	2	3	6	2	4
Fishing fee related to M	Z=4.96	Baqueiro et al. 2003	2	1	2	2	4
Breeder biomass (SSB) or other proxies	NA	NA	2	0	0	0	0
Seasonal migrations	It is dispersed in fine, thick sand and tepetate to feed outside the reproductive period between 5 and 50 m deep	Baqueiro et al. 2003 CEDO 2016 field data	2	3	6	1	2
Schools/ Aggregations	They make small aggregations in areas of fine-medium sand for reproduction	Loaiza et al. 2012 CEDO 2012	2	1.5	3	1	2
Morphology that affects the capture	None, only behavior	Loaiza et al. 2012	2	2.5	5	1	2
Survival after capture and release	High survival after release	Fishing and personal observations	2	1	2	3	6
Convenience / value of the fishery	High value for the local fishing sector of diving ($ 11 pesos / kg current beach price) *.	*Com. Pers. Alejandrino Madueño June 2016	2	2	4	3	6
Impact of the fishery on habitats essential for fish (EFH) or habitats in general for non-target species.	With trap the impact is relative depending on the loss of traps and when it is with diving (little), the impact is low due to the manual capture of the bottom with compressor diving	CEDO 2012, Report for Sustainable Fisheries Partnership 16 July 2012	2	2	4	2	4
Final result Susceptibility					1.91		
Final result Vulnerability			1.15				

Table 4S (cont.)

Brown crab							
**Productivity Attributes**	**Data**	**Source**	**Weight**	**Attribute score**	**Weighted attribute score**	**Data quality score**	**Weighted data quality score**
r (Population growth)	T(2B)= 1 year	Valdez-Ornelas 2011	2	2	4	2	4
Maximum age	4 a 5 year	CEDO 2012	2	3	6	2	4
Maximum size	177 mm	CEDO 2012	2	3	6	2	4
Growth coefficient Von Bertalanffy (k)	k=0.9 (1/year)	Hernández & Arreola Lizárraga et al. 007	2	3	6	3	6
Estimated natural mortality (M)	1.19 year-1	Molina et al. 2006	2	3	6	3	6
Measured fertility	1'400,000 eggs/female	CEDO 2012	2	3	6	1	2
Strategy reproductive	Aggregation reproductive in Cerro Prieto, Santo Tomas and Desemboque Strategy "r"	CEDO 2012	2	3	6	2	4
Pattern recruitment	Annual from start from summer until autumn	CEDO 2012	2	2	4	2	4
Age of maturity	1 year	CEDO 2012	2	1	2	1	2
Average trophic level	3.05	WORMS and CEDO	2	2	4	1	2
Final result productivity					2.5		

Table 4S (cont.)

Brown crab							
**Productivity Attributes**	**Data**	**Source**	**Weight**	**Attribut score**	**Weighted attribute score**	**Data quality score**	**Weighted data quality score**
Management Strategy	Fishing permits, exploitation rate of 50% of the biomass and minimum catch size of 115 mm. Recommends a fishing ban from May 1 to June 30 and the first week of July.	Carta Nacional Pesquera (2012). CEDO 2012	2	2	4	2	4
Superposition of areas	Approximately 30% occurs in the Corridor	Management plan Brown crab 2014	2	1	2	1	2
Geographical concentration	The majority of the population is located in the Upper Gulf, encompassing the states of Sonora, BC and BCS	Brown crab Management plan 2014	2	2	4	1	2
Vertical superposition	In general it is found in sandy and protected areas, in the Corridor from El Borrascoso to Desemboque de Caborca	Brown crab management plan 2014	2	3	6	1	2
Fishing rate related to M	Total mortality Z is 1.06 per year	Brown crab management plan 2014	2	3	6	1	2
Biomass of reproducers (SSB) u other proxies	The biomass is not determined, approximately 33%	Valdez Ornelas 2011	2	2	4	3	6
Migrations seasonal	It is "buried" in sand and conchales after its reproduction (2 months)	CEDO 2012	2	1	2	2	4
Schools / Aggregation	It is added to start the reproduction although segregation by sex has been seen, they have closed in reproductive season	Management plan Brown crab 2014	2	2	4	1	2
Morphology that affects the capture	The traps are selective and have low FAC	Report for Sustainable Fisheries Partnership 16 july 2012. COBI/CDO	2	3	6	2	4
Survival after capture and release	They must return to the sea the M and F with boys and ovigerous mass	Management plan 2014	2	2	4	1	2
Convenience / value of	National and	Management	2	3	6	1	2

the fishery	international markets such as EU, Japan and Korea are described	plan Brown crab 2014					
Impact of the fishery on habitats essential for fish (EFH) or habitats in general for non-target species	Not reported but can affect if the traps are lost as they continue to work for more than 6 months	POP-CONAPESCA 2014	2	2	4	1	2
Final result susceptibility					2.17		
Final result vulnerability			1.27				

Table 4S (cont.)

Guitarfish							
**Productivity Attributes**	**Data**	**Source**	**Weight**	**Attribut score**	**Weighted attribute score**	**Data quality score**	**Weighted data quality score**
r (Population growth)	Annual growth rate 0.189	Valdez-Ornelas et al 2007	2	1	2	1	2
Maximum age	16 years / 11 years (M) / 16 years (F)	Fishbase /Downton 2007	2	2	4	1	2
Maximum size	119 cm TL (M-non-sexed); 170 cm TL (F) / 156 cm H 142/114 cm F/M 101/66 cm F/M	Fishbase / Baxter 1966 Pacific Coast Downton 2007 Pacific coast Márquez 2005 California of Gulf Downton 2007 Pacific coast Márquez 2005 Gulf of California	2	2	4	1	2
Coefficient of growth Von Bertalanffy (k)	0.1 / 0.166 F 0.24 M /Annual growth 0.189	Fishbase / Downton 2007 / Valdez-Ornela et al 2007	2	2	4	1	2
Natural mortality estimated (M)	M=0.27 (annual)	Downton 2007	2	2	4	1	2
Measured fertility	Absolute Fertility min 6; max 16/8 embryos / 6 embryos	Fishbase Downton 2007 Márquez-Farías 2005	2	1	2	1	2
Reproductive strategy	Ovoviviparous gestation lasts 12 months. Reproductive aggregations Strategies "k"	Fishbase Downton 2007 Márquez-Farías 2005	2	1	2	2	4
Recruitment pattern	A seasonal peak per year Annual-Summer	Fishbase Downton 2007 Márquez-Farías 2005	2	3	6	1	2
Age of maturity	7 years F - 6 years 3 years	Downton 2007 Márquez-Farías 2007	2	1	2	1	2
Average trophic level	3.5 - 3.7	Fishbase	2	1	2	2	4
Final result productivity					1.6		

Table 4S (cont.)

Guitarfish							
**Productivity Attributes**	**Data**	**Source**	**Weight**	**Attribute score**	**Weighted attribute score**	**Data quality score**	**Weighted data quality score**
Management Strategy	The 2010 National Fishing Charter mentions not issuing new permits for commercial catch, only renewal or replacement. Maintain annual catches above 15,000 t. (Pacific) Gulf of California a closed season from May 1 to July 31.	National Fishing Charter (2010). Data sheet Coastal sharks (2010)	2	3	6	4	8
Superposition of areas	The corridor area is the most productive area of the Gulf of California Contains a good proportion of the stock biomass of the northern Gulf of California	Downton 2007 Márquez 2005	2	2	4	2	4
Geographical concentration	It's found commonly in temperate waters throughout the coast of the Pacific Ocean, It is distributed from San Francisco, California, to the Gulf of California, Mexico	Castro-Aguirre, 1965 in Downton 2007	2	2	4	3	6
Vertical superposition	Demersal species that migrates from shallow areas of less than 1m to deep areas up to 91.5m	IUCN Downton 2007	2	1	2	2	4
Fishing fee related to M	Z = 0.33-0.36	Downton 2007	2	1	2	1	2
Breeder biomass (SSB) or other proxies	Annual	Downton, 2007	2	1	2	2	4
Seasonal migrations	The offsprings remain in in protected bays and estuaries during the first year of lifetime. From their second year onwards performed migrations of zones deep to shallow areas (reproduction)	Downton, 2007	2	1	2	2	4

Schools/Aggregions	Form aggregations reproductive in Bays and estuaries for Its reproduction	Downton, 2007	2	1	2	2	4
Morphology that affects the capture	The rostrum becomes entangled in the net and continuous circular movements lead to the whole body being entangled	Downton, 2016.	2	2	4	3	6
Survival after capture release	Good as they are entangled for a short time	Downton, 2016.	2	2	4	3	6
Convenience / value of the fishery	It presents a high commercial value among the group of elasmobranchs since sometimes they pass the meat as a tope shark	Marquez, 2011	2	2	4	2	4
Impact of the fishery on habitats essential for fish (EFH) or habitats in general for non-target species	The impact varies depending on the season and the fishing area, but being a network tends to capture other species whether elasmobranch or others that remain in the network	MIA, 2012	2	1	2	2	4
Final result susceptibility					1.58		
Final result Vulnerability			1.52				

Table 4S (cont.)

Pacific Angel shark							
**Attributes of Susceptibility**	**Data**	**Source**	**Weight**	**Attribute score**	**Weighted attribute score**	**Data quality score**	**Weighted data quality score**
r (population growth)	0.056	Cailliet et al 1992	2	1	2	1	2
Maximum age	35 years<br>11 years (male) / 16 years (female)	Fishbase<br>Downton 2007	2	1	2	1	2
Maximum size	152 cm TL H / not sexed<br>118 M	Fishbase<br>Natanson 1984	2	2	4	1	2
Growth coefficient von Bertalanffy (k)	0.188	Natanson, 1986.	2	2	4	1	2
Estimated natural mortality (M)	M=0.2	Cailliet et al 1992	2	2	4	1	2
Measured fertility	6 embryos<br>~ 20% offspring survive maturity<br>9-11 oocytes per female	Natanson and Cailliet 1986, Ebert 2003<br>Fishbase	2	0.2	0.4	2	4
Reproductive strategy	Reproductive aggregations. Strategists "k" Ovoviviparous.	Villavicencio et al. 1998	2	2	4	1	2
Recruitment pattern	Recruitment pattern	Natanson and Cailliet 1986, Ebert 2003	2	3	6	2	4
Age of maturity	8-13 years	Fishbase	2	1	2	1	2
Average trophic level	4.14	Fishbase<br>Escobar Sanchez et al. 2011	2	1	2	1	2
Final Result Productivity					1.52		

Table 4S (cont.)

Pacific angel shark

Attributes of Susceptibility	Data	Source	Weight	Attribute score	Weighted attribute score	Data quality score	Weighted data quality score
Management Strategy	The 2010 National Fishing Charter mentions not issuing new permits for commercial catch, only renewal or replacement. Maintain annual catches above 15,000 t. (Peaceful). Gulf of California a closed season from May 1 to July 31.	National Fishing Charter (2010). Coastal Sharks info sheet (CEDO 2010)	2	3	6	4	8
Superposition of areas	The area of the corridor is the most productive area of the Gulf of California, contains a good proportion of the biomass of the northern Gulf of C stock.	Bizzarro et al 2007	2	2	4	2	4
Geographical concentration	Eastern Pacific southeast of Alaska to the Gulf of California and Costa Rica south of Chile	Florida Museum	2	2	4	2	4
Vertical Superposition	In coastal areas from 6 to 43 m deep and in the Gulf of California up to 183 m	Compagno et al. 1995. Gaida, 1997	2	1	2	2	4
Fishing fee related to M	M=0.2 F=0.22, Z=0.42	Calliet, 1992	2	2	4	1	2
Breeder biomass (SSB) or other proxies	Each female each female produces 2.6 H breeders	Calliet, 1992	2	1	2	2	4
Seasonal migrations	Migrates from the Pacific to the Gulf of California in summer to reproduce	Escobar Sanchez, et al 2011	2	1	2	1	2
Schools/Aggregations	It has reproductive aggregations in summer	Escobar Sanchez et al 2011	2	1	2	1	2
Morphology that affects the capture	The gill slits facilitate its entanglement	fishers Desemboque	2	2	4	4	8
Survival after capture and release	Low survival because entanglement in this species limits gill movements	Downton, 2016.	2	1	2	4	8
Convenience / value of the fishery	High commercial value among the elasmobranch group	Bizzarro et al 2007	2	2	4	2	4
Impact of the fishery on essential fish habitats (EFH)	The impact varies depending on the season and the fishing area, but	MIA, 2012	2	1	2	2	4

or habitats in general	being a network tends to capture other species whether elasmobranchs or others that remain in the network			
Final result Susceptibility				
Final result Vulnerability		1.59	1.58	

Table 4S (cont.)

Banded guitarfish

Susceptibility attributes	Data	Source	Weight	Attribute score	Weighted attribute score	Data quality score	Weighted data quality score
r (population growth)	0.07 taken from *Rhinobatus productus*	Downtown 2007 Farrugia et al. 2016	2	1	2	3	6
Maximum age	14 or 15 years old family Rhinobatidae	Abascal Monroy et al 2012	2	2	4	3	6
Maximum size	83 cm M / not sexed 97cm H / 90 cm	Fishbase Blanco Parra et al. 2009	2	2	4	2	4
Growth coefficient von Bertalanffy (*k*)	0.21	Fishbase	2	2	4	2	4
Estimated natural mortality (M)	0.3 / 0.31	Hoening, 1983/Jensen, 1997 (Calculated by Downton en 2016)	2	2	4	2	4
Measured fertility	Average of 7.13 ± 2.9 embryos per female	Blanco Parra 2009	2	0.2	0.4	2	4
Reproductive strategy	K species life strategy	Blanco Parra 2009	2	2	4	1	2
Recruitment pattern	It is known that this species has an important breeding season (embryonic development, births and copulation) during spring and summer in Sonora	Blanco Parra 2009	2	3	6	2	4
Age of maturity	1.9 years / 3.5 years	Fishbase	2	2	4	2	4
Average trophic level	3.6	Fishbase	2	1	2	2	4
Final score Productivity			10		1.72		

Table 4S (cont.)

Banded guitarfish							
**Attributes of Susceptibility**	**Data**	**Source**	**Weight**	**Attribute score**	**Weighted attribute score**	**Data quality score**	**Weighted data quality score**
Management Strategy	The 2010 National Fishing Charter mentions not issuing new permits for commercial catch, only renewal or replacement. Maintain annual catches above 15,000 t. (Peaceful). Gulf of California fishing ban from May 1 to July 31.	National Fishing Charter (2010). Coastal Sharks CEDO (2010)	2	3	6	4	8
Superposition of areas	The area of the corridor is the most productive area of the Gulf of California, contains a good proportion of the biomass of the northern Gulf of C stock.	Bizzarro et al 2007	2	2	4	2	4
Geographical concentration	California to Mazatlan, including the Gulf of California	Blanco Parra 2009	2	2	4	2	4
Vertical Superposition	Coastal species found generally on rocky reefs, from the intertidal zone to depths of 69 m and occasionally on sandy bottoms	Blanco Parra 2009	2	1	2	2	4
Fishing fee related to M	Being a species of the same family as R productus we could use the same parameter Z = 0.36 (annual) have a similar occurrence period	Downton 2007	2	2	4	4	8
Breeder biomass (SSB) or other proxies	Each female produces 3.5 H breeders	Blanco-Parra et al, 2009.	2	1	2	2	4
Seasonal migrations	From February to July it approaches shallow areas for reproduction	Blanco Parra 2009	2	1	2	1	2
Schools / Aggregation	It has reproductive aggregations in summer	Blanco Parra 2009	2	1	2	1	2
Morphology that affects the capture	The rostrum is entangled and continues circular movements to free itself with what the body is entangled.	Downton, 2016.	2	2	4	4	8
Survival after capture	They can have good	Downton,	2	1	2	4	8

and release	survival in short-time meshes.	2016.					
Convenience / value of the fishery	High commercial value only in some communities (BSJ)	Bizzarro et al 2007	2	3	6	2	4
Impact of the fishery on habitats essential for fish (EFH) or habitats in general for species not target	For this species there is no data, however its behavior is equal to that of the Guitar. The impact varies depending on the season and the fishing area, but being a network tends to capture other species whether elasmobranch or others that remain in the network	Fisherman BSJ/ Downton 2016 MIA, 2012	2	1	2	2	4
**Final result Susceptibility**							
**Final result Vulnerability**			1.44		1.67		

Table 4S (cont.)

Brown smooth-hound shark

Susceptibility attributes	Data	Source	Weight	Attribute score	Weighted attribute score	Data quality score	Weighted data quality score
r (population growth)	r=0.175 to *Mustelus schmitti*	Corts 2007	2	1	2	3	6
Maximum age	14 years F / 9 years M<br>13 years	Méndez 2008<br>Fishbase	2	2	4	1	2
Maximum size	100 cm	Chabot et al. 2015./ Cortes 2002	2	2	4	2	4
Growth coefficient von Bertalanffy (k)	0.14 F, 0.28 M<br>0.23	Méndez 2008<br>Fishbase	2	2	4	1	2
Estimated natural mortality (M)	M= 0.30<br>0.32	Frisk et al. 2005<br>Fishbase	2	2	4	1	2
Measured fertility	1-21 offspring in the Northern Gulf of California<br>3-5 offspring	Chabot et al. 2015<br>Fishbase	2	1	2	2	4
Reproductive strategy	K species life strategy	Fishbase	2	2	4	1	2
Recruitment pattern	Annual recruitment 4 years M / 6 years F	Fishbase	2	3	6	2	4
Age of maturity	Annual recruitment 4 years M / 6 years F	Méndez 2008	2	1	2	2	4
Average trophic level	3.6	Fishbase	2	1	2	2	4
Final result Productivity					1.7		

Table 4S (cont.)

Attributes of Susceptibility	Data	Source	Weight	Attribute score	Weighted attribute score	Data quality score	Weighted data quality score
Brown Smooth-hound shark							
Management Strategy	The 2010 National Fishing Charter only mentions not issuing new permits for commercial catch, only renewal or replacement. Maintain annual catches> 15,000 t. (Peaceful). Gulf of California: Fishing ban May 1 - July 31	Carta Nacional Pesquera (2010). Sheet Shore Sharks (CEDO 2010)	2	3	6	4	8
Superposition of areas	The area of the Corridor is the most productive in the Gulf of California, contains a high proportion of the stock biomass in the N Gulf	Bizzarro et al 2007	2	2	4	2	4
Geographical concentration	Northern California to the Gulf of California and Peru	Guía FAO 2005 Fish Base	2	2	4	2	4
Vertical superposition	Intermareal to 200m	Chabot et al. 20151	2	1	2	2	4
Fishing fee related to M	Z = 1.9 mortality conditioned by the availability of the resource (seasonal)	Danemann & Escurra 2008	2	1	2	2	4
Breeder biomass (SSB) or other proxies	Each female produces 10 F breeders	Silva-Santos, 2012	2	1	2	2	4
Seasonal migrations	Make migrations from March to July corresponding to the spring / summer season. Performs temporary migratory movements	Silva-Santos, 2012	2	1	2	2	4
Schools / Aggregations	In the spring months they are added in low areas outside San Jorge Bay and in areas near Puerto Lobos but not so shallow	Comp pers fishers PLO	2	1	2	4	8
Morphology that affects the capture	None, only behavior, are enmeshed by movement	Coms. Pers fishers PLO & BSJ Downton, 2016	2	2	4	4	8
Survival after capture and release	It has low survival when entangled because it needs to maintain water flow in gills.	Downton, 2016.	2	1	2	4	8

Convenience / value of the fishery	The annual catch in the GC is 12,643 t, of which the genus Mustelus constitute 79.6% of the catches	Marquez-Frias 2000 Bizzarro et al 2007	2	1	2	2	4
Impact of the fishery on habitats essential for fish (EFH) or habitats for non-target species	The impact varies depending on the season and fishing area, but being a network tends to capture other species whether elasmobranchs or other remaining network	MIA, 2012	2	1	2	2	4
Final result Susceptibility					1.42		
Final result Vulnerability			1.37				

Table 4S (cont.)

Gulf croaker							
**Attributes of Susceptibility**	**Data**	**Source**	**Weight**	**Attribute score**	**Weighted attribute score**	**Data quality score**	**Weighted data quality score**
r (population growth)	Time doubling population 1.4 - 4.4 years r= 0.175	Fishbase	2	2	4	3	6
Maximum age	~ 17 years	Román-Rodríguez, M. J., 2000	2	2	4	1	2
Maximum size	40.0 cm TL M/ Not sexed 507 mm L 420 LP, 1163 gr	Fishbase Román-Rodríguez 2000	2	2	4	1	2
Growth coefficient von Bertalanffy (k)	K=0.42 year K=0.51	Fishbase Marta Roman 2000	2	3	6	1	2
Estimated natural mortality (M)	0.4103	Román-Rodríguez 2000	2	3	6	1	2
Measured fertility	180,000 eggs - 39.5 cm TL H M. undulates	Hildebrand & Cable 1930 en Sink et al. 2010.	2	2	4	3	6
Reproductive strategy	Life strategy “r”	Román Rodríguez, M. J., 2000	2	1	2	2	4
Recruitment pattern	The spawning process gradually ends in summer (July, August)	Román-Rodríguez, M. J., 2001	2	2	4	2	4
Age of maturity	1.7 years Size 400-410 mm	Fishbase Román-Rodríguez 2000	2	3	6	2	4
Age of medium maturity	3.5	Fishbase	2	2	4	2	4
Final score Productivity					2.2		

Table 4S (cont.)

Attributes of Susceptibility	Data	Source	Weight	Attribute score	Weighted attribute score	Data quality score	Weighted data quality score
Gulf croaker							
Management Strategy	The National Fishing Charter 2010 (page 53) only specifies to take measures if the catch decreases of 2000 tons of curvinas-berrugatas and chano together in Sonora, only mentions management with permission of general scale.	The National Fishing Charter 2010	2	3	6	4	8
Superposition of areas	The area of the corridor is the most productive area of the Gulf of California, endemic and contains a good proportion of the stock biomass	CEDO 2017	2	2	4	3	6
Geographical concentration	Reported as endemic to the Gulf of California Others consider it is distributed from the Colorado River Delta to the coast of Guerrero in southern Mexico	Roman 1997 FishBase, Flores et al. 1999	2	1	2	2	4
Vertical Superposition	Restricted distribution, endemic to the Gulf of California	Aragón-Noriega et al. 2010	2	2	4	2	4
Fishing fee related to M	Z=0.45	Roman 2000	2	1	2	1	2
Breeder biomass (SSB) or other proxies	NA	Pers. Obs. Loaiza	2	3	6	4	8
Seasonal migrations	After the reproductive period (April, May) larvae and juveniles are kept in the channels of the Colorado River delta from the months of January to September, when spawning populations of the central and southern Gulf of	Román Rodríguez 2000	2	1	2	2	4

	California are kept near coastal lagoons and bays.						
Schools / Aggregation	April, May and June is the reproductive period of the chano, it is found in large concentrations and it is easier to capture it	Inquiry PANGAS 2007	2	1	2	2	4
Morphology that affects the capture	They are usually enmeshed by gills and fins, in addition to their behavior	Pers. Obs. Loaiza	2	2	4	2	4
Survival after capture and release	Low survival, gillnets remain in operation for several hours	Obs pers. Loaiza	2	1	2	2	4
Convenience / value of the fishery	One of the 5 most important fisheries in the Upper Gulf of California; it represents 27% of the fish catch.	Aragón-Noriega et al. 2010. Management program RBAGCyDC 2007.	2	2	4	1	2
Impact of the fishery on habitats essential for fish (EFH) or habitats in general for non-target species	Se obtiene una pesca incidental de varias especies de peces e invertebrados de aproximadamente 1:0.32	Aragón-Noriega et al. 2010. Management program RBAGCyDC 2007.	2	2	4	2	4
Final result Susceptibility					1.75		
Final result Vulnerability			1.1				

Table 4S (cont.)

Gold spotted sand bass							
**Attributes of Susceptibility**	**Data**	**Source**	**Weight**	**Attribute score**	**Weighted attribute score**	**Data quality score**	**Weighted data quality score**
r (population growth)	4.4 years r=calculated 0.175	Aburto Oropeza et al 2008	2	2	4	3	6
Maximum age	24 years, average longevity 18 years	Aburto Oropeza et al 2008	2	2	4	2	4
Maximum size	71 cm LT<br>47 cm L std	FishBase Pondella et al. 2001	2	2	4	2	4
Growth coefficient von Bertalanffy (k)	0.1<br>0.115	FishBase Pondella et al. 2001	2	1	2	2	4
Natural mortality estimated (M)	0.49<br>0.177	Pondella et al. 2011 Hoening 1983 (Calculated by Downton in 2016)	2	2	4	2	4
Measured fertility	For the Gulf of California, P. maculatofaciatus is 10,300 oocytes per spawn and it is known to have a long spawning period, P. auroguttatus.	Lluch Cota 1995	2	2	4	2	4
Reproductive strategy	Does not care for eggs<br>Lays eggs in open water or spreads on the substrate	Aburto Oropeza et al 2008	2	2	4	2	4
Recruitment pattern	Annual/summer	Aburto Oropeza et al 2008	2	1	2	2	4
Age of maturity	2 years	Aburto Oropeza et al 2008	2	2	4	2	4
Average trophic level	3<br>4.2	Aburto Oropeza et al 2008 FishBase	2	1	2	2	4
Final Result Productivity					1.7		

Table 4S (cont.)

Gold spotted sand bass

Attributes of Susceptibility	Data	Source	Weight	Attribute score	Weighted attribute score	Data quality score	Weighted data quality score
Management Strategy	The National Fisheries Charter 2010 only mentions taking the necessary measures in case Baja California, Sonora and Sinaloa catches decrease by 200 t per year, mentions that the management is with perch permits for Sonora (it is known that this is not the case) and recommends evaluations in every way.	La Carta Nacional Pesquera 2010. Info sheet Gulf Coney, Grouper and Verdillo (Serranidae)	2	3	6	4	8
Superposition of areas	The area of the corridor is the most productive area of the Gulf of California, contains a good proportion of the biomass of the northern Gulf of C stock.	Cedo 2017	2	2	4	3	6
Geographical concentration	From the Pacific coast in Baja California, the entire Gulf of California, Mexico.	Fish Base	2	2	4	2	4
Vertical superposition	Juvenile shallow to deep zones. Adults in rocky patches between 18 and 155 m	Aburto Oropeza et al 2008	2	2	4	2	4
Fishing fee related to M	Fishing mortality is 1.3 times greater than the natural F=0.531 Z=0.708	Danemann & Escurra, 2007	2	3	6	2	4
Breeder biomass (SSB) or other proxies	NA	Pers. Obs. Loaiza	2	3	6	4	8
Seasonal migrations	It has reproductive aggregations from March to May, period of greatest capture of the	Danemann & Escurra, 2007	2	1	2	2	4

	species						
Schools / Aggregation	Reproductive gre gations from March to May, period of greatest catch of the species	Danemann & Escurra, 2007	2	1	2	2	4
Morphology that affects the capture	They are usually enmeshed by their gills and fins, in addition to their behavior	Obs pers. Loaiza	2	1	2	2	4
Survival after capture and release	Low survival, decompression of swim bladder and spaces with gas	Pers. Obs. Loaiza	2	3	6	2	4
Convenience / value of the fishery	It presents high value in the fishery although not as much as the Baqueta	Obs pers. Loaiza	2	2	4	2	4
Impact of the fishery on habitats essential for fish (EFH) or habitats in general for non-target species	It is not known to impact the habitat, it is extracted with shoring	Pangas 2012. CEDO 2017	2	1	2	2	4
Final result Susceptibility					2		
Final result Vulnerability			1.64				

Aburto–Oropeza O., Erisman B., Valdez–Ornelas C., Danemann G. 2008. Serránidos de importancia comercial del Golfo de California: Ecología, pesquerías y conservación. Cienc. Conserv. (1): 1–23.

Balmori A., Torre J., Rojo M. y Loaiza R. 2012.La fauna de acompañamiento en la pesquería de Jaiba en el Golfo de California (Sonoa y Sinaloa). Informe para Sustainable Fisheries Partnership 16 julio 2012. 24 pp.

Blanco-Parra M.P., Márquez-Farías F. y Galván-Magaña F. 2009. Fishery and morphometric relationships of the banded guitarfish, *Zapteryx exasperata* (Elasmobranchii, Rhinobatidae), from the Gulf of California, Mexico. Pan-american J. Aquatic Sci. 4(4): 456-465.

Blue Water Media. https://chesapeakebay.noaa.gov/fish-facts/blue-crab

Cailliet, G.M., Chabot, C.L., Nehmens, M.C. & Carlisle, A.B. 2016. *Squatina californica*. The IUCN Red List of Threatened Species 2016: e.T39328A80671059. http://dx.doi.org/10.2305/IUCN.UK.2016-2.RLTS.T39328A80671059.en. Downloaded on 20 January 2017.

Cailliet G., Mollet H. y Nathanson L. 1992. Growth and demography of the Pacific Angle Shark (*Squatina californica*), based upon tag returns off California. Marine and Freshwater Research. Vol 43. 1313-30.

Chabot C. L., Espinoza M., Mascareñas-Osorio I. y Rocha-Olivares A. 2015. The effect of biogeographic and phylogeographic barriers on gene flow in the brown smoothhound shark, *Mustelus henlei*, in the northeastern Pacific. Ecology and Evolution. Vol 5(8): 1585-1600

Chao, L., Espinosa, H. & van der Heiden, A. 2010. *Micropogonias megalops*. The IUCN Red List of Threatened Species 2010: e.T183538A8130848. http://dx.doi.org/10.2305/IUCN.UK.2010-3.RLTS.T183538A8130848.en. Downloaded on 20 January 2017.

Cudney-Bueno R. y Turk-Boyer P. 1998. Pescando entre mareas del alto Golfo de California. Una guía sobre la pesca artesanal, su gente y sus propuestas de manejo. Centro Intercultural de Estudios de Desiertos y Océanos CEDO, A.C. Puerto Peñasco, Sonora, México. 175 pp

Cudney-Bueno R., Prescott R., & Hinojosa-Huerta O. 2008. The Black Murex Snail, *Hexaplex nigritus* (Mollusca, Muricidae), in the Gulf of California, Mexico: I. Reproductive Ecology and Breeding Aggregations. Bulletin of Marine Science, 83(2), 285-298.

Cudney-Bueno R. and Rowell K. The black murex snail, *Hexaplex nigritus* (Mollusca, Muricidae), in the Gulf of California, México: II. Growth, longevity, and morphological variations with implications for management of a rapidly declining fishery. Bulletin of Marine Science, 83(2): 299-313 pp

DOF, 2014. NORMA Oficial Mexicana NOM-039-PESC-2003, Plan de Manejo Pesquero de Jaiba (Callinectes spp.) de Sinaloa y Sonora. Publicado en el Diario Oficial de la Federación el miércoles 24 de junio de 2014.

Downton-Hoffmann C. A. 2007. Biología del pez guitarra *Rhinobatos productus* (Ayres, 1856), en Baja California Sur, México. Centro Interdisciplinario de Ciencias Marinas. Tesis de doctorado. La Paz, B.C.S. México. 194 pp.

Echeverria F., Otero V., Cornejo F. y Rodríguez J. 2002. WSSV y ciclo reproductivo de muda en el camarón blanco *Litopenaeus vannamei*. El mundo acuícola 8 (1), 43-46

Escobar-Sánchez O., Galván-Magaña F., Abitia-Cárdenas L.A. 2011 Trophic level and isotopic composition of δ 13C and δ 15N of *Squatina californica* in the southern Gulf of California, Mexico. J Fish Aquat Sci 6(2):141–150

Farrugia T.J., Márquez-Farías F., Freedman R.M., Lowe C.G, Smith W.D. y Bizzarro J.J. 2016. *Pseudobatos productus*. The IUCN Red List of Threatened Species 2016: e.T60171A104004394. Downloaded on 12 December 2016 [http://www.iucnredlist.org/details/60171/0]

Florida Museum. https://www.flmnh.ufl.edu/fish/discover/species-profiles/squatina-californica

Frisk M. G. y Miller T. J. 2005. Life Histories and Vulnerability to Exploitation of Elasmobranchs: Inferences from Elasticity, Perturbation and Phylogenetic Analyses. Journal of Northwest Atlantic Fishery Science. Vol 35: 27-45

Froese, R. and D. Pauly. Editors. 2016. FishBase. World Wide Web electronic publication. www.fishbase.org, version (10/2016).

Gaida I. H. 1997. Population Structure of the Pacific Angel Shark, *Squatina californica* (Squatiniformes: Squatinidae), around the California Channel Islands. American Society of Ichthyologist and Herpetologists (ASIH). Vol 1997 (4) 738-744 pp

Góngora-Gómez A. M., García-Ulloa Gómez M., Dominguez-Orozco A. L. y Camacho-Sánchez F. Y. 2011. Aspectos reproductivos cuantitativos del caracol murex negro, Hexaplex migritus (Phillipi, 1845) en condiciones de laboratorio. Ciencia y Mar 2011, XV (44): 31-34

Hernández L. y Arreola-Lizárraga J. A. 2007. Estructura de tallas y crecimiento de los cangrejos *Callinectes arcuatus* y *C. bellicosus* (Decapoda: Portunidae) en la laguna costera Las Guásimas, México Revista de Biología Tropical, vol. 55, núm. 1. pp. 225-233

Labastida-Che A y Núñez-Orozco A. L. 2015 Parámetros bioógicos de *Callinectes arcuatus* y *Callinectes bellicosus* en el sistema lagunar Mar Muerto, Oaxaca-Chiapas, México. Ciencia Pesquera. Número especial 23:27-34 pp

Loaiza-Villanueva R., Castañeda-Fernández de Lara V., Pérez-Valencia S., Sánchez-Cruz A., & Martínez-Tobar I. 2009. Monitoreo Poblacional de Jaiba *Callinectes* spp. en la Costa Norte de Sonora en el Corredor Bahia Adhair-Desemboque de Caborca, 2008-2009, Región Puerto Peñasco Sonora. Informe Técnico, Centro Intercultural de Estudios de Desiertos y Océanos, A.C. (CEDO). Puerto Peñasco, Sonora, México. 19 pp

López-Mártinez J., López-Herrera L., Valdez-Holguín J. E. y Rábago-Quiroz C. H. 2014. Population dynamics of the swimming crabs *Callinectes* (Portunidae) components pf shrimps bycatch in the eastern coast of the Gulf of California. Revista de biología marina y oceanografía. Vol. 49. No. 1. Valparaíso abr. 17-29 pp

Lluch Cota D. B. 1995. Aspectos reproductivos de la cabrilla arenera, *Paralabrax maculatofasciatus* (Piesces: Serranidae) en Bahía Magdalena-Almejas, Baja California Sur, México. Universidad Autónoma de Baja California Sur. Tesis de Maestría. 186 pp.

Márquez–Farías J.F. 2005. Gillnet mesh selectivity for the shovelnose guitarfish (*Rhinobatos productus*) from fishery–dependent data in the artisanal ray fishery of the Gulf of California, Mexico. J. Northwest Atl. Fish. Sci. (35): 443–452.

Márquez-Farías J. F. 2007. Demografía del pez guitarra*, Rhinobatos productus* (Ayres, 1854), del Golfo de California. Centro de Investigaciones Biológicas del Noroeste, S.C. Tesis de doctorado. La Paz, B. C. S. 147 pp

Natanson L. y Cailliet G. M. 1986. Reproduction and Development of the Pacific Angel shark. *Squatina californica*, off Santa Barbara, California. American Society of Ichthyologists and Herpetologist (ASIH). Vol 1986(4). 987-994 pp.

Pondella II D. J., Allen L. G., Rosales-Casian J. A. and Hovey T. E. 2001. Demographic parameters of Golden Spotted Rock Bass *Paralabrax auroguttatus* from the Northern Gulf of California. American Fisheries Society. 130: 686-691 pp

Prescott R. and Cudney-Bueno R. 2008. Mobile 'reefs' in the northeastern Gulf of California: aggregations of black murex snails *Hexaplex nigritus* as habitat for invertebrates. Marine Ecology Progress Series. Vol. 367: 185-192 pp

Román-Rodríguez M. J. 2000. Estudio poblacional de chano norteño, *Micropogonias megalops* y la curvina golfina *Cynoscion othopterus* (Gilbert) (Pisces:Sciaenidae), especies endémicas del alto Golfo de California, México. Instituto del Medio Ambiente y Desarrollo Sustentable del Estado de Sonora. Informe final SNIB-CONABIO proyecto No. L298. Méxic D.F.

"Sink T., Strange R., Lochmann R. y Gatlin III D. 2010. Hatchery methods and natural, hormone-implant induced and synchronized spawning of captive Atlantic croaker (*Micropogonias undulatus*) Linnaeus 1766. Aquaculture 307: 35-43.

Valdez-Ornelas V. M., Aburto-Oropeza O., Torreblanca-Ramirez E., Danemann G. D. y Vidal-Talamantes R. 2007. Capítulo 5. Recursos Pesqueros. Bahía de Los Ángeles: recursos naturales y comunidad: línea base 2007. Instituto Nacional de Ecología. 1480 pp

Valenzuela-Quiñonez F. 2009 Hábitos alimenticios del pez guitarra Rhinobatos productus en el alto Golfo de California. Centro de Investigaciones Biológicas del Noroeste, S.C. Tesis de Maestría. LA Paz, B.C.S. México. 94 pp

Table 5S. Size frequency data. Interval (Int.) and frequency (Fq.)

Black murex		Pink Murex		Brown crab		Guitarfish		Gold spotted sand bass		Gulf croaker		Smooth brown-hound	
Int.	Fq.	Int.	Fq.	Int.	Fq.	Int.	Fq.	Int.	Fq.	Int.	Fq.	Int.	Fq.
5.25	0	3.26	0	4.76	0	38.5	0	20.5	0	29.5	0	38.5	0
5.5	1	3.5	1	5.04	4	39.5	1	22	1	30	1	40	1
5.75	0	3.74	0	5.32	0	40.5	1	23.5	0	30.5	0	41.5	0
6	0	3.98	0	5.6	1	41.5	0	25	0	31	0	43	0
6.25	0	4.22	0	5.88	0	42.5	1	26.5	1	31.5	0	44.5	0
6.5	0	4.46	0	6.16	1	43.5	1	28	1	32	4	46	4
6.75	0	4.7	0	6.44	0	44.5	3	29.5	3	32.5	0	47.5	0
7	0	4.94	0	6.72	2	45.5	2	31	2	33	6	49	6
7.25	0	5.18	0	7	2	46.5	7	32.5	2	33.5	0	50.5	6
7.5	2	5.42	3	7.28	4	47.5	5	34	8	34	8	52	11
7.75	0	5.66	1	7.56	3	48.5	6	35.5	10	34.5	0	53.5	8
8	1	5.9	0	7.84	8	49.5	10	37	12	35	10	55	18
8.25	1	6.14	10	8.12	10	50.5	10	38.5	19	35.5	0	56.5	2
8.5	2	6.38	7	8.4	20	51.5	7	40	23	36	5	58	9
8.75	1	6.62	3	8.68	17	52.5	13	41.5	10	36.5	0	59.5	7
9	12	6.86	4	8.96	33	53.5	4	43	17	37	18	61	13
9.25	3	7.1	1	9.24	129	54.5	4	44.5	14	37.5	0	62.5	8
9.5	7	7.34	1	9.52	188	55.5	4	46	29	38	9	64	32
9.75	6	7.58	0	9.8	143	56.5	0	47.5	19	38.5	0	65.5	7
10	25	7.82	6	10.08	348	57.5	7	49	20	39	12	67	12
10.25	11	8.06	7	10.36	401	58.5	12	50.5	7	39.5	0	68.5	13
10.5	15	8.3	15	10.64	371	59.5	19	52	9	40	5	70	32
10.75	13	8.54	9	10.92	394	60.5	5	53.5	3	40.5	0	71.5	12
11	117	8.78	9	11.2	338	61.5	17	55	7	41	15	73	14
11.25	33	9.02	33	11.48	474	62.5	20	56.5	3	41.5	0	74.5	7
11.5	48	9.26	20	11.76	507	63.5	1	58	4	42	8	76	11
11.75	18	9.5	31	12.04	503	64.5	11	59.5	0	42.5	0	77.5	4
12	98	9.74	23	12.32	398	65.5	19	61	1	43	14	79	20
12.5	31	9.98	30	12.6	206	66.5	17	62.5	1	43.5	0	80.5	5
12.5	51	10.22	128	12.88	278	67.5	3	64	0	44	12	82	3
12.75	28	10.46	46	13.16	273	68.5	12	65.5	0	44.5	0	83.5	3
13	231	10.7	87	13.44	227	69.5	6	67	0	45	6	85	8
13.25	27	10.94	35	13.72	260	70.5	16	68.5	0	45.5	0	86.5	3
13.5	52	11.18	166	14	160	71.5	9	70	0	46	6	88	3
13.75	25	11.42	33	14.28	205	72.5	6	71.5	0	46.5	0	89.5	4
14	129	11.66	49	14.56	26	73.5	7	73	0	47	5	91	3
14.25	28	11.9	12	14.84	143	74.5	8	74.5	0	47.5	0	92.5	2
14.5	27	12.14	155	15.12	67	75.5	10	76	0	48	3	94	3
14.75	14	12.38	4	15.4	38	76.5	0	77.5	0	48.5	0	95.5	2
15	41	12.62	18	15.68	29	77.5	1	79	0	49	3	97	3
15.25	5	12.86	0	1596	11	78.5	3	80.5	0	49.5	0	98.5	0
15.5	3	13.1	26	16.24	15	79.5	0	82	0	50	0	100	0
15.75	2	13.34	0	16.52	3	80.5	0	83.5	0	50.5	0	101.5	4
16	10	13.58	5	16.8	1	81.5	0	85	0	51	0	103	5
16.25	0	13.82	0	17.08	1	82.5	1	86.5	0	51.5	0	104.5	4
16.5	0	14.06	7	17.36	1	83.5	0	88	0	52	0	106	4
16.75	1	14.3	0	17.64	0	84.5	0	89.5	0	52.5	0	107.5	1
17	7	14.54	1	17.92	0	85.5	0	91	0	53	0	109	0
17.25	0	14.78	0	18.2	0	86.5	0	92.5	0	53.5	0	110.5	1
17.5	1	15.02	2	18.48	0	87.5	0	94	0	5<br>4	1	112	1
17.75	0	15.26	0	18.76	0	88.5	0	95.5	0	54.5	0	113.5	0
18	3	15.5	0	19.04	1	89.5	0	97	0	5<br>5	1	115	1
18.25	0	15.74	0	19.32	0	90.5	2	98.5	2	55.5	0	116.5	4
-	-	15.98	0	-	-	91.5	1	100	0	-	-	118	0
-	-	16.22	1	-	-	92.5	1	-	-	-	-	-	-
-	-	16.46	0	-	-	-	-	-	-	-	-	-	-
**TOTAL**	**11114(**		**989**		**6344**		**293**		**228**		**152**		**324**

Table 6S. Froese sustainability indicators. Percentage of composition of the catch with individuals at maturity, 10% ± of optimal length (Lopt) and mega-reproducers. The goals for each of these indicators are indicated in parentheses.

		Indicator		
**Species**	**No. samples**	**Mature (100%)**	**10%± L_{opt} (100%)**	**Mega reproducers (<40%)**
Black murex	1140	72.3%	39.6%	58.3%
Pink murex	989	52%	10.6%	31.5%
Brown crab	6344	32.2%	1.7%	4.9%
Guitarfish	293	87.4%	18.8%	3.4%
Pacific Angel shark		-	-	-
Banded guitarfish	28	-	-	-
Gold spotted sand bass	228	9.2%	16.7%	4.4%
Gulf croaker	152	99.3%	154.6%	119.1%
Brown smooth-hound	324	73.8%	37.7%	13.9%

Table 7S. Criteria used for SEASALT, management-based attribute analysis.

Attribute		Criteria for evaluation
**Secure**	Tenure length of fishing rights	VERY HIGH = Quota / area transfer (lease / sale) is done through a formalized mechanism, and transfers can be made in real time through established (often digital) markets.
		HIGH = Transfer (lease / sale) of quota / area is done through the formalized mechanism that is clear and accessible, but cannot be done in real time.
		AVERAGE = Transfer (lease / sale) of quota / area is formalized (in law or management plan), but transfers are difficult to execute.
		LOW = Transfer (lease / sale) of fees / area is done by informal mechanisms (not written in the laws or in the management plans).
		NO = Transferability does not exist.
		0 = Not enough information to determine the status of the indicator
	Renewal of fishing rights	5 = There is a clear renewal process, which has been well established as evidenced by a strong history of renewal.
		4 = There is a clear renewal process, but there is no renewal history.
		3 = There is no clear process for renewal, but there is a presumption of renewal through historical evidence.
		2 = There is no clear process for renewal, and renewal is uncertain or unlikely.
		1 = Clearly stated that there should be no renewal of privileges.
		0 = There is not enough information to determine the performance of the indicator.
	Ability to defend rights legally	5 = There are clear legal provisions to defend privileges, and there is historical evidence of their legal defense.
		4 = There are clear legal provisions to defend privileges, but historical evidence of defense cannot be found.
		3 = There are legal provisions to defend privileges but they are unclear.
		2 = The recognition of privileges is informal (that is, it is not reflected in laws or legislation).

		1 = There is no recognition of formal or informal privileges and / or there are no permits or licenses.<br><br>0 = There is not enough information to determine the performance of the indicator.
**Exclusive**	Rights are clearly defined by the allocation of quotas	5 = There is a formal and individual quota allocation for all target species in the fishery; These are established with a clear and transparent process that is communicated in a timely manner.<br><br>4 = There is an allocation of formal and individual actions for all target species of the fishery; but the processes may not be transparent.<br><br>3 = There is an allocation of formal and individual actions for all target species of the fishery, but without allocation to individual entities (eg fishermen, cooperatives, vessels). The process is not transparent.<br><br>2 = There is only informal allocation (not defendable by law) of quotas.<br><br>1 = There is no share allocation or any other RBM instrument (formally or informally) in place.<br><br>0 = There is not enough information to determine the performance of the indicator.
	Penalties for violation of privilege by third parties	5 = All violations are punishable and are frequently prosecuted, resulting in high compliance.<br><br>4 = Some violations are punishable, but only a few are prosecuted, resulting in an average enforcement.<br><br>3 = Few violations are punishable, but are rarely prosecuted, resulting in low compliance.<br><br>2 = Violations are punishable, but there is no prosecution, resulting in no compliance<br><br>1 = Violations of the privilege are not punishable.<br><br>0 = There is not enough information to determine the performance of the indicator.
	Effect of new entrants on existing fees	5 = Provisions are implemented such that new operators do not have an effect on the individual fees of existing participants (eg fees that are reserved for new entrants)<br><br>4 = The provisions implemented are clear about whether new entrants are welcomed, these will have an effect on the individual fees of current entrants, but historical evidence shows that new operators have not affected existing fees.

		3 = The provisions in place are clear about whether new entrants will have an effect on individual participant fees for current entrants, and it is not known whether new entrants will have an effect on existing fees.<br>2 = The provisions implemented are clear that if new entrants are welcomed, they will have an effect on the individual fees of existing entrants, but there has been no evidence that new operators will reduce existing fees.<br>1 = New entrants are free to enter the program, and it is clearly stated that accommodating them will lead to a redistribution of fees, resulting in a reduction of individual fees for existing participants.<br>0 = There is not enough information to determine the performance of the indicator.
**All sources**	Assignment of arrivals and discards by catch	5 = There is little or no bycatch or discards, or if discards and / or bycatches are a problem, participants receive an individual allocation for specific species and discards and / or bycatch.<br>3 = Participants will receive a species-specific allowance, which is adjusted for estimated discards (discard limit and / or bycatch is not explicit), or participants have an entire discard and / or discard fishery limit. bycatch.<br>1 = Participants receive an allowance for specific species, but there is no limit on discards and / or bycatch.<br>0 = There is not enough information to determine the performance of the indicator.
	Controls on fishing mortality incorporate other fleets or sport / recreational users who fish the same stocks	5 = Controls for fishing mortality incorporate arrivals and discards from the sports sector and / or other fleets; it is based on accurate and complete data or there is evidence that the fishery has no interaction with these sectors.<br>4 = Controls for fishing mortality incorporate arrivals and discards from the sports sector and / or other fleets; it is based on moderate quality data.<br>3 = Controls on fishing mortality incorporate arrivals and discards from the sports sector and / or other fleets; estimated with very low quality data and limited availability<br>2 = Controls on fishing mortality do not take into account the impacts of other resource users and these other users are on a small or unknown scale.<br>1 = Controls on fishing mortality take into account the impacts of other resource users, but these other users are large-scale.

		0 = There is not enough information to determine the performance of the indicator.
**Scaled**	Population is managed by a single well-coordinated and accountable unit	5 = The entire population of the target species is managed under a coordinated unit.<br>4 = A part of the population of the target species is managed under catch quotas or access rights, while the rest is managed by a different unit (for example, council, state, country, community), and there is coordination between the bodies management.<br>3 = Part of the population of the target species is managed under catch quotas or access rights, while the rest is managed by a different unit (council, state, country), and there is little or no coordination between management bodies. .<br>2 = Part of the population of the target species is managed under catch quotas or access rights, while the rest of the population have no management.<br>1 = The catch quota is not used for the population of the target species.<br>0 = There is not enough information to determine the performance of the indicator. "
	Political, cultural, social and economic differences between the fishing groups within the program are integrated into the design	5 = The program is designed taking into account the political, cultural, social, and economic characteristics of each group within the program, or the program was explicitly designed to include only similar fishing users, therefore there are no significant differences between participants in the fishery.<br>4 = There are differences between the fishing groups within the program, they have been identified, and there is an action plan to integrate the differences of the groups within the program, or there are no significant differences between the participants in the fishery of populations under management.<br>3 = There are differences between the groups of fishermen in the program, they have been identified and recognized, however there is no technical capacity (or political will) to integrate them into the management program.<br>2 = There are differences between the fishing groups within the program and have been identified, but the administrators decided to leave them out of the fisheries management program.<br>1 = There are differences between the fishing groups within the program, but they are not recognized or integrated into the design of the program.

		0 = There is not enough information to determine the performance of the indicator.
**Accountable**	Participatory resource management	5 = Resource management decisions are made jointly by organized local actors, especially fishermen, and the authority under a co-management regime.<br>4 = There are formal mechanisms for local actors, particularly fishermen, to express their opinion on resource management with some influence on decision-making.<br>3 = There are formal mechanisms in place for local actors, particularly fishermen, to express their opinion on resource management; however, they have no influence on decision making.<br>2 = Local actors, particularly fishermen, are informed, but do not participate in the decisions that are being made regarding the resource.<br>1 = Local actors, particularly fishermen, are neither involved nor informed of the decisions made regarding the resource.<br>0 = There is not enough information to determine the performance of the indicator
	There are mechanisms to enforce community regulations, standards and / or agreements	5 = Each incident is sanctioned, in a way that discourages any future infraction OR compliance is high based on other forms of accountability (eg social institutions).<br>4 = Compliance is frequent, and penalties are sufficient to deter future violations OR compliance is average based on other forms of accountability.<br>3 = Compliance is frequent, but sanctions are insufficient OR compliance is irregular based on other forms of accountability.<br>2 = Compliance mechanisms are in place but used infrequently (due to limited resources, multiple layers of bureaucracy, corruption) OR compliance is low based on other forms of accountability.<br>1 = There is no compliance mechanism in place or there is no compliance with community rules, regulations and / or agreements.<br>0 = There is not enough information to determine the performance of the indicator.
	Monitoring systems are up and running	5 = Strong systems exist to monitor fishermen: where they fish and what they catch at all times (eg, observer coverage ≈100%).

		4 = There are monitoring efforts that track and record the activity of the fishing participants with high frequency and adequate reliability.<br>3 = There are monitoring efforts, the information has adequate reliability, but the monitoring and recording is carried out irregularly.<br>2 = There are some monitoring efforts; however, the monitoring is irregular and the information has low reliability.<br>1 = There are no monitoring systems.<br>0 = There is not enough information to determine the performance of the indicator.
**Limited**	Best available science is used to limit fishing mortality	5 = The best available science is used to calculate a fishing mortality limit, is incorporated into fisheries management, and has the opportunity to be adapted over time as the biology and populations of target species change.<br>4 = The best available science is used to calculate a fishing mortality limit, it is incorporated into fisheries management, but adaptive management is not implemented.<br>3 = Scientific information is used to calculate a fishing mortality limit; however, this limit is NOT actively incorporated into fisheries management.<br>2 = There is a limit on fishing mortality; however, it is not clear that it was established using the best available science.<br>1 = There is no control that limits fishing mortality.<br>0 = There is not enough information to determine the performance of the indicator.
**Transferability**	Not applicable	Not applicable

Table 8S. Fishery management restrictions included in the base scenario of the Atlantis ecosystem model simulations

Restrictions to age structure	Regulatory instrument
Shrimp	NOM-002-SAG/PESC-2013
Oyster	DOF 16/03/94
Brown crab	NOM-039-PESC-2003
Gulf corvina	NOM-063-PESC-2005
Mojarra	NOM-016-PESC-1994
Sharks	NOM-026-PESC-2006
**Effort restrictions**	
Shrimp	NOM-002-PESC-2006
Sea cucumber	Advertisement DOF 16/03/1994
Gulf corvina	NOM-063-PESC-2005
Sharks	NOM-026-PESC-2006
Sport fishing	NOM-017-PESC-1994
Industrial shrimp trawling	NOM-002-SAG/PESC-2013
Industrial tuna fishery	DOF 16/07/2014
Mojarra	NOM-016-PESC-1994

Figure 1S. Size frequency analysis. The graph shows the age of first maturity (Lm, orange line), the optimal length (Lopt, line and gray interval), and the theoretical maximum length (Linf, green line) per species.

A. Black murex snail

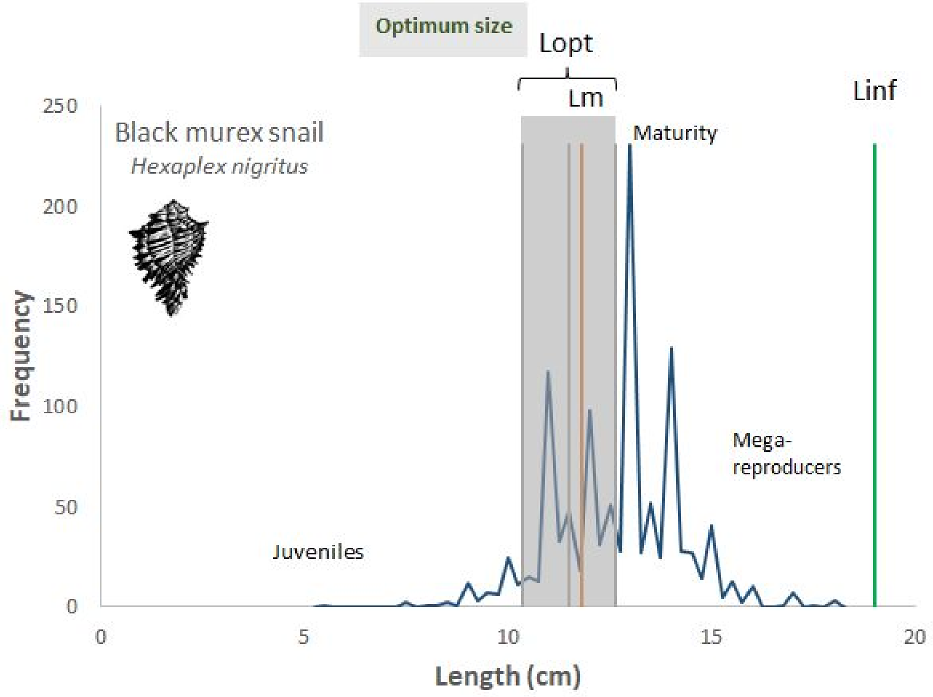

B. Pink murex snail

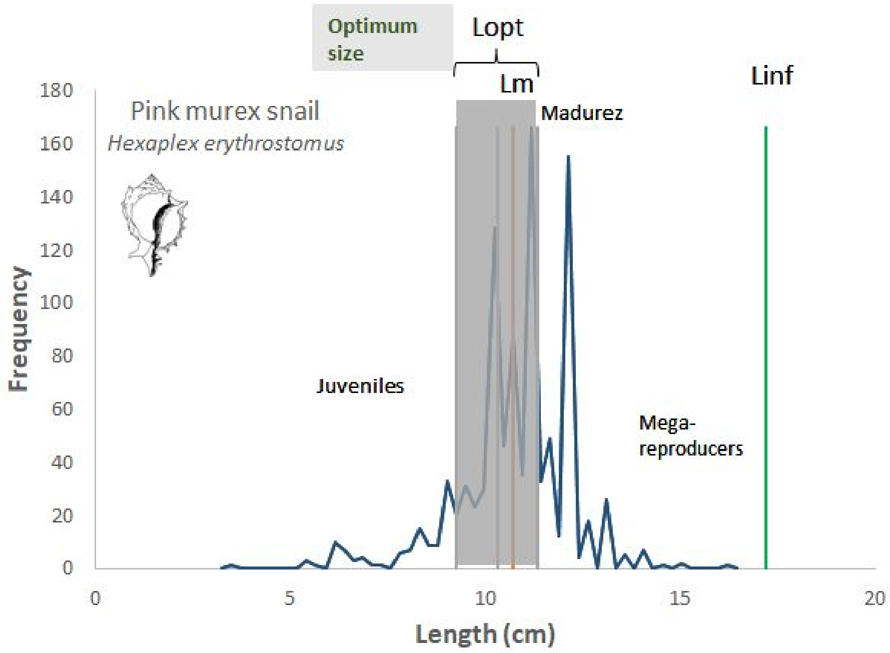

C. Brown crab

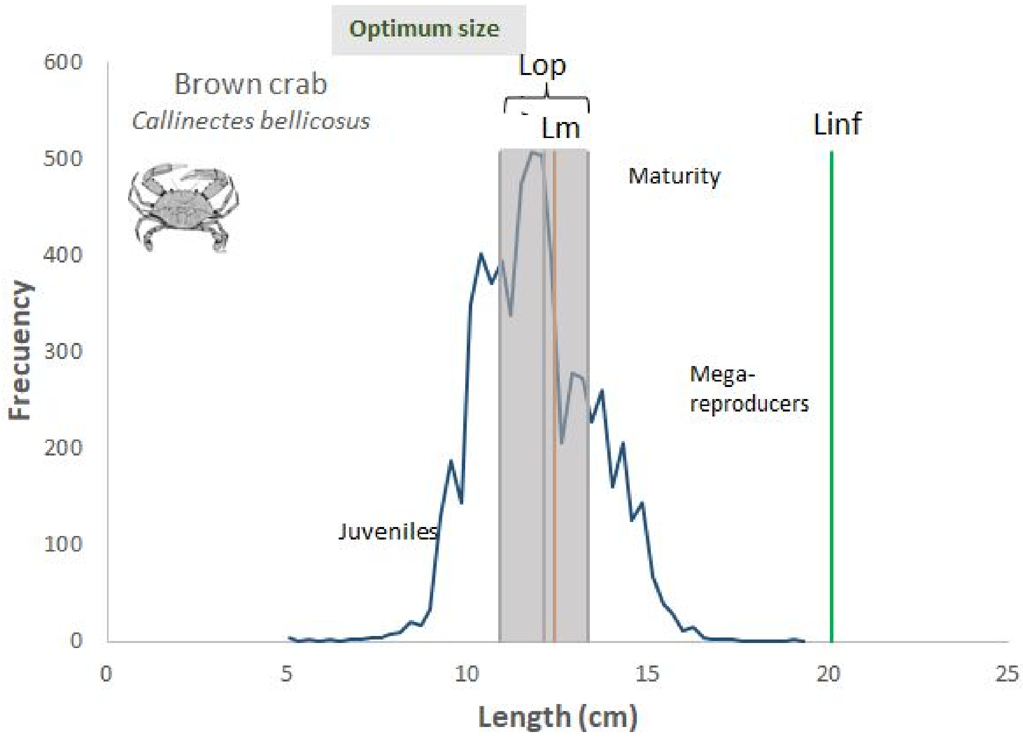

D. Guitarfish

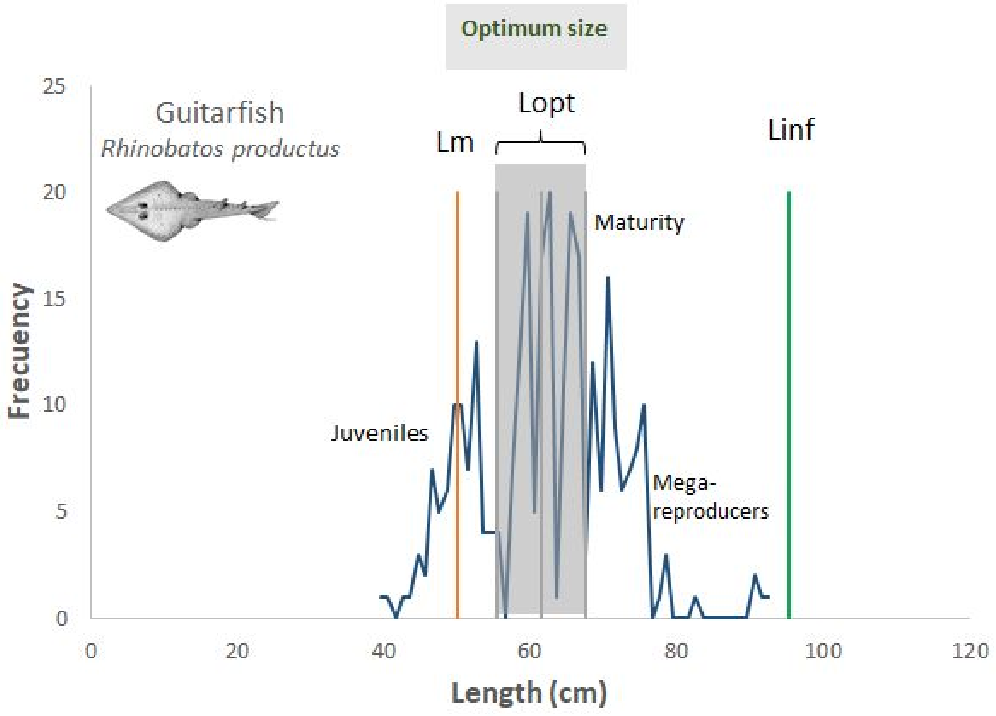

E. Banded guitarfish

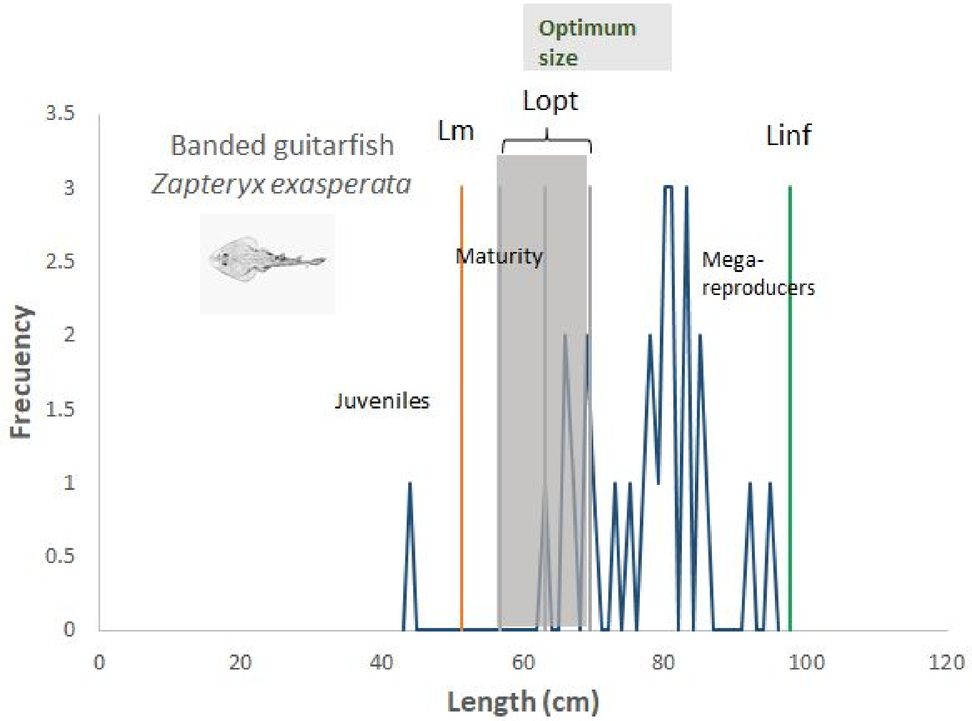

F. Gold-spotted sand bass

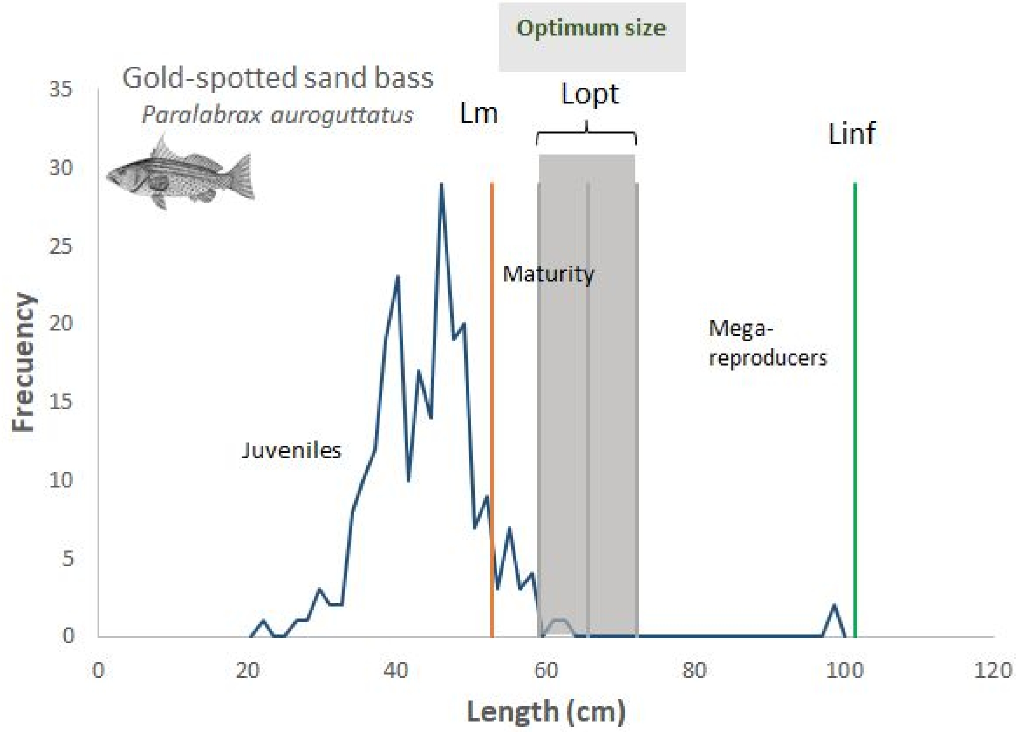

G. Gulf Croaker

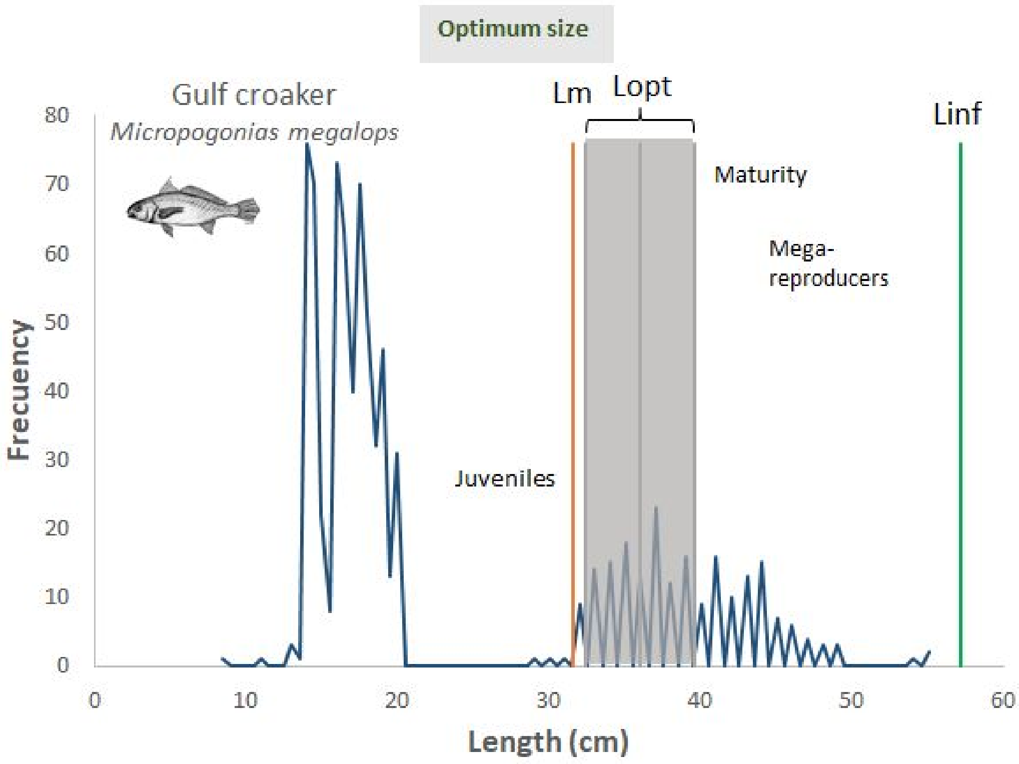

H. Brown smooth-hound shark

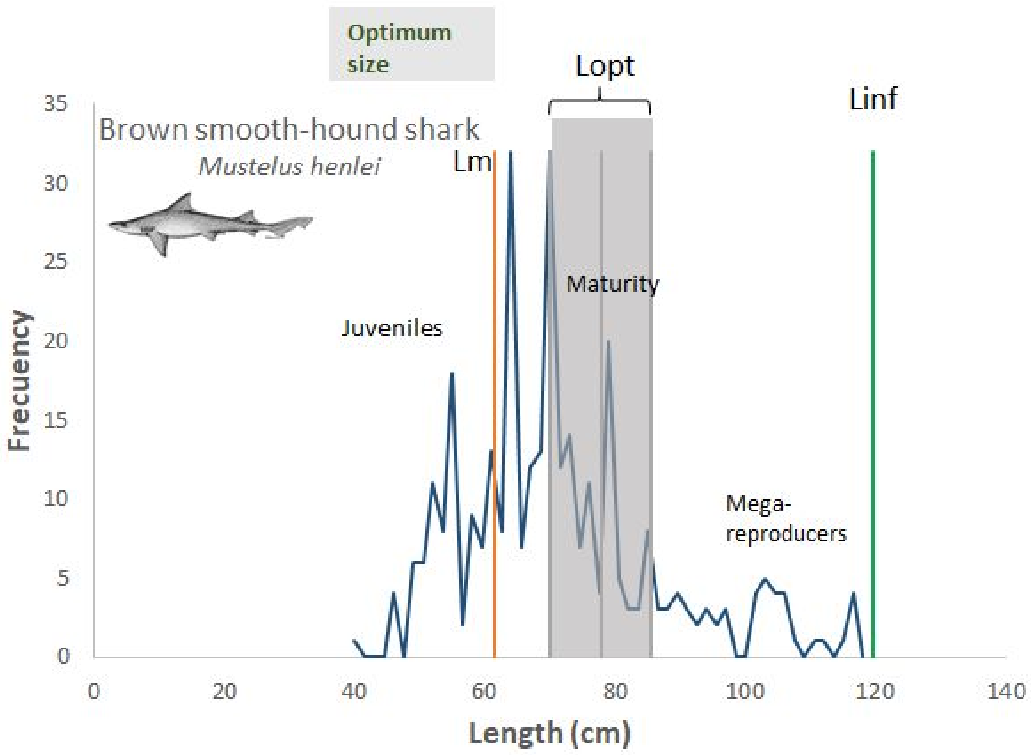

Figure 2S. Species considered for reduction in fishing mortality in the scenarios for the trade-off analysis.

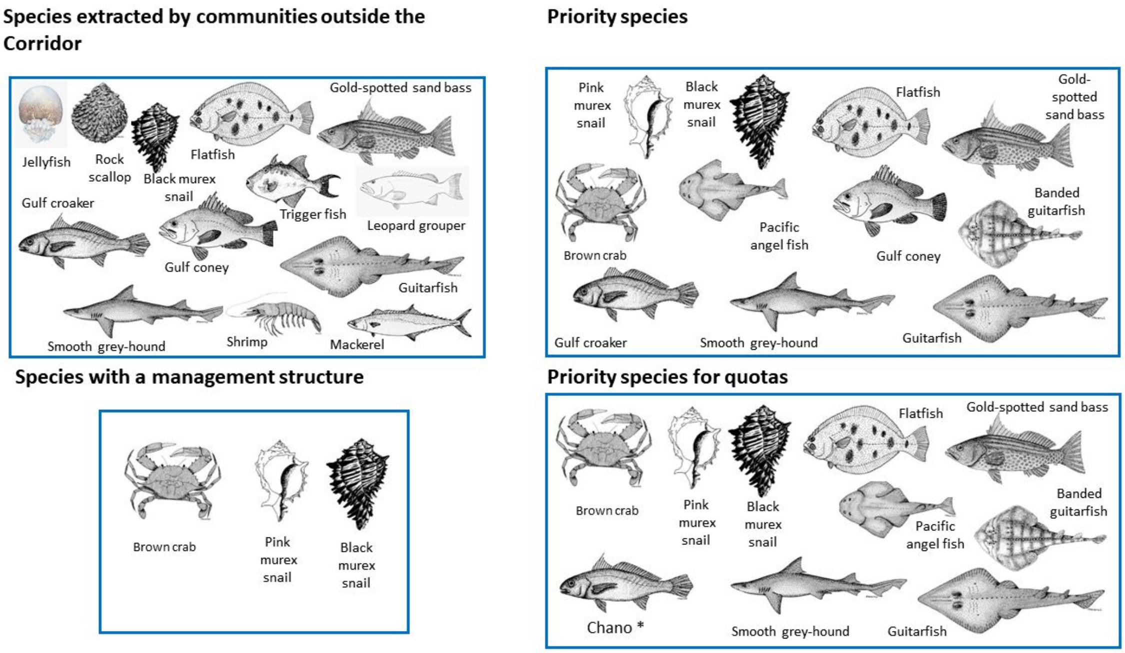

Figure 3S. Natural Protected Areas considered in the Atlantis Ecosystem Model.

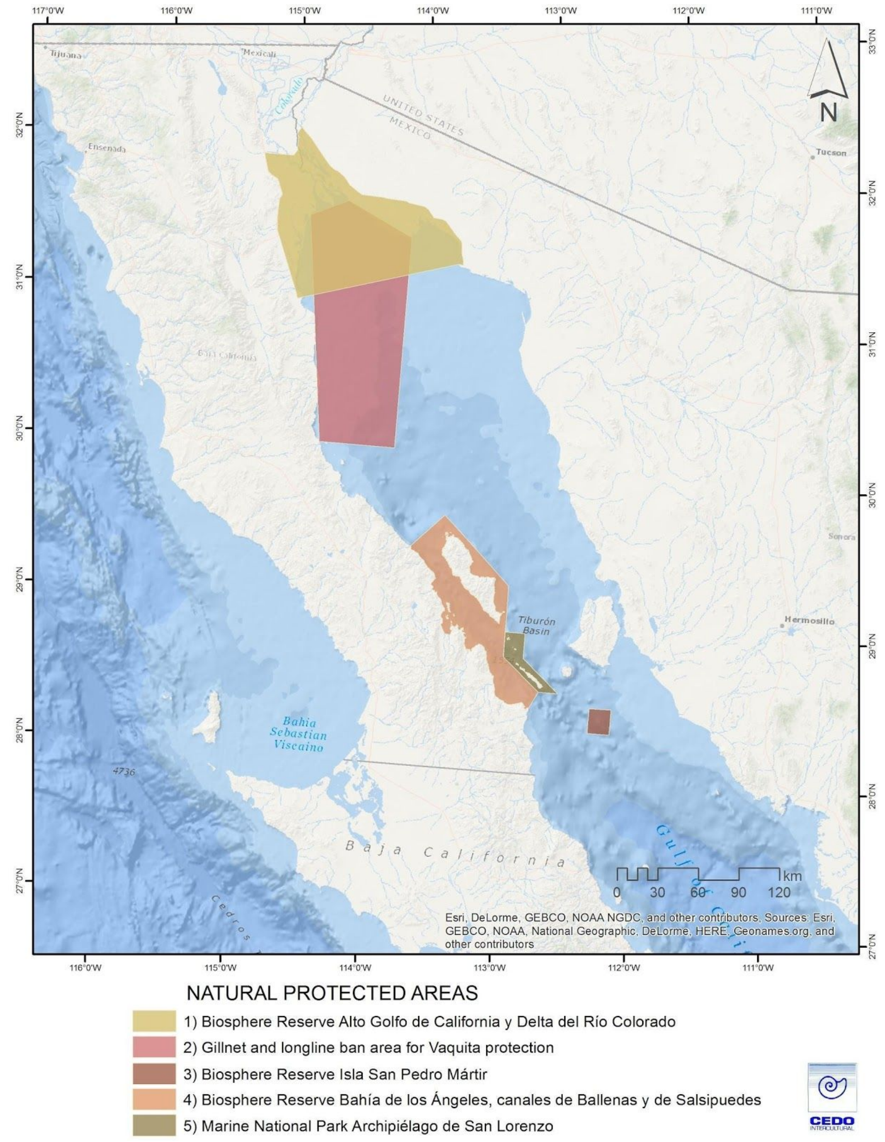

Figure 4S. Illustrative diagram of a radar graph used to show alternative benefits between each indicator for the scenarios in the trade-off analysis. A. Explains how to read the radar graph. B. Shows an example with all indicators used.

A.

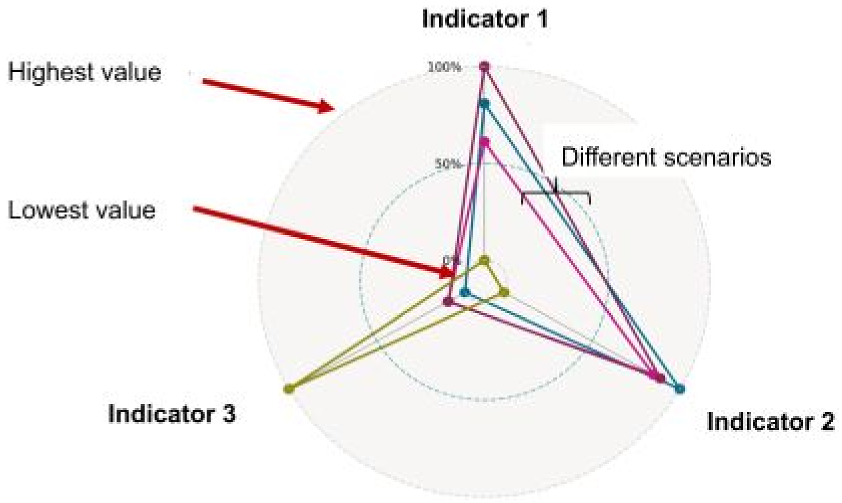

B.

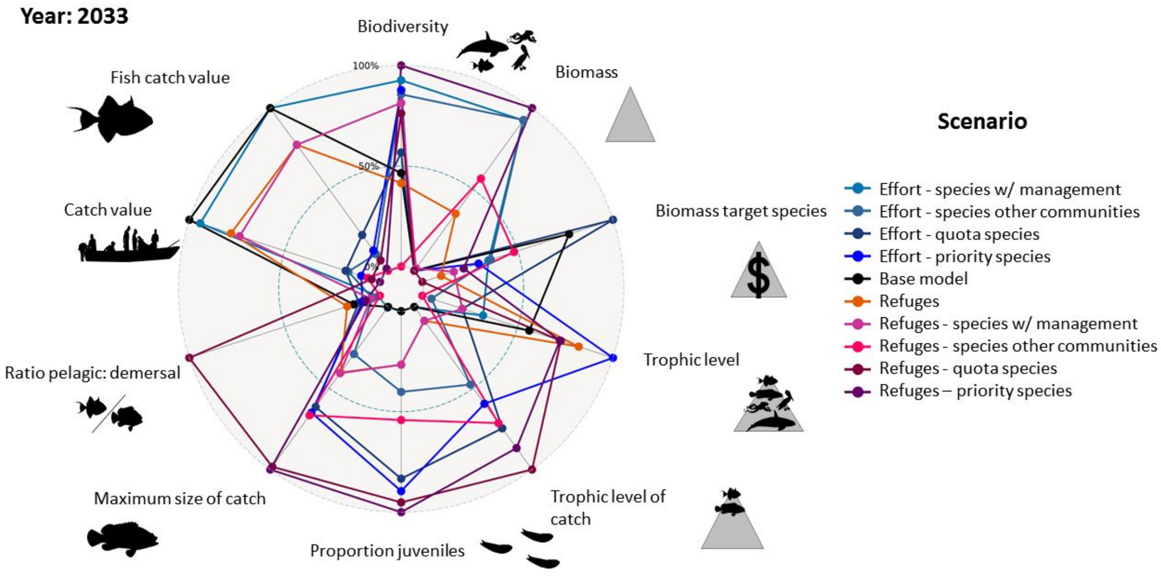

Supplement: Supplementary file 1 [file mmc1.pdf]
